# Mucoactive Agents in Muco-Obstructive Lung Diseases: A Critical Reappraisal of Pharmacological Effects and Clinical Outcomes

**DOI:** 10.3390/ph19050681

**Published:** 2026-04-27

**Authors:** Domenico Larobina, Giorgia Franzino, Fabiana Tescione, Michela Abrami, Domenico Tierno, Alice Biasin, Federica Tonon, Anna De Nes, Marta Maggisano, Paola Confalonieri, Annalucia Carbone, Marco Confalonieri, Gabriele Grassi, Sante Di Gioia, Mario Grassi, Massimo Conese

**Affiliations:** 1 Institute of Polymers, Composites and Biomaterials, National Research Council of Italy, P. le E. Fermi 1, I-80055 Portici, Italy; giorgiafranzino@cnr.it (G.F.); fabiana.tescione@cnr.it (F.T.); 2 Department of Engineering and Architecture, University of Trieste, Via Valerio 6/A, I-34127 Trieste, Italy; mikystars@hotmail.com (M.A.); mario.grassi@dia.units.it (M.G.); 3 Department of Medicine, Surgery and Health Sciences, University of Trieste, Strada di Fiume 447, I-34149 Trieste, Italy; domenico.tierno@units.it (D.T.); alice.biasin@units.it (A.B.); federica.tonon@asugi.sanita.fvg.it (F.T.); ggrassi@units.it (G.G.); 4 Pulmonology Unit, Department of Medical Surgical and Health Sciences, Hospital of Cattinara, University of Trieste, I-34149 Trieste, Italy; anna.denes@studenti.units.it (A.D.N.); s312173@stud.units.it (M.M.); paola.confalonieri@asugi.sanita.fvg.it (P.C.); mconfalonieri@units.it (M.C.); 5 Department of Clinical and Experimental Medicine, University of Foggia, I-71122 Foggia, Italy; annalucia.carbone@unifg.it (A.C.); sante.digioia@unifg.it (S.D.G.)

**Keywords:** mucoactive agents, muco-obstructive lung diseases, airway mucus, oxidative stress, anti-inflammatory agents

## Abstract

Muco-obstructive lung diseases, such as chronic obstructive pulmonary disease (COPD), cystic fibrosis (CF), and bronchiectasis, are characterized by the accumulation of highly viscoelastic mucus that compromises mucociliary clearance and fosters infection and inflammation. Mucoactive therapy, encompassing both true mucolytics and non-cleaving agents, seeks to restore airway patency by altering mucus structure, hydration, and transport properties, yet its clinical impact remains variable. This narrative review provides a critical reappraisal of the pharmacological actions and therapeutic outcomes of the main mucolytic agents: N-acetylcysteine (NAC), erdosteine, carbocisteine, bromhexine, ambroxol, and dornase alfa. Beyond their classical role in reducing mucus viscosity, these drugs exhibit pleiotropic effects, including antioxidant, anti-inflammatory, and immunomodulatory activities. Specifically, for thiol-based compounds, the action consists of breaking the disulfide bonds that stabilize the mucin network; for carbocisteine, it lies in modulating mucin glycosylation and chloride transport. Ambroxol and bromhexine act by stimulating surfactant secretion and enhancing mucociliary clearance. Finally, dornase alfa exerts an enzymatic effect on extracellular DNA, a key contributor to the tenacity of mucus in cystic fibrosis. Clinical evidence indicates that NAC and erdosteine can reduce exacerbation rates in COPD, carbocisteine shows benefit with prolonged administration, and dornase alfa remains a cornerstone in CF management. However, therapeutic efficacy is constrained by heterogeneous mucus composition, pharmacokinetic limitations, and disease-specific variability. A key interpretative message is that clinical benefit appears greatest when the dominant biophysical determinant of mucus pathology is specifically targeted, supporting a transition from broad disease-label prescribing to mechanism-informed, phenotype-aware mucolytic therapy. Emerging strategies, such as agents targeting mucin–DNA interactions and advanced inhalation delivery systems, promise improved specificity and durability. By integrating mechanistic insights with clinical data, this review underscores the need for personalized mucolytic therapy and innovative approaches to overcome current challenges in managing muco-obstructive lung diseases.

## 1. Introduction

Mucoactive agents are drugs that facilitate the clearance of airway secretions through a variety of mechanisms, including modification of mucus structure, hydration, and transport. Within this broad category, true mucolytics are defined as agents that directly cleave or depolymerize mucus constituents (e.g., disulfide-rich mucins or extracellular DNA), whereas other mucoactive agents act primarily as expectorants or mucoregulators by enhancing secretion, surfactant release, or mucociliary transport [1,2,3]. They are used in the treatment of muco-obstructive lung diseases, i.e., in diseases that involve the production of “thick” mucus, like in the case of cystic fibrosis (CF), chronic obstructive pulmonary disease (COPD), bronchiectasis and asthma. Their mechanism of action depends on the specific interactions they target at the molecular level. Indeed, the macroscopic viscoelastic properties of mucus arise from a complex network of interactions among its components, all of which contribute to defining its overall behavior [4,5]. The choice of mucolytic depends on the underlying disease, severity of symptoms and patient tolerance [6]. Before listing the different mucolytic agents, it is therefore essential to provide an overview of the composition, structure, and interactions that characterize the properties of the mucus, particularly its viscoelastic behavior. A detailed description of the experimental techniques used to characterize mucus structure and rheology is provided in Appendix A (mucus characterization), together with the search strategy. The experimental techniques include macro-rheology (bulk viscoelastic properties), micro-rheology (local heterogeneity and mesh size via particle tracking), and Low-Field (LF)-NMR (network architecture and hydration state).

A healthy adult produces approximately 2 L of airway mucus every 24 h to keep the tracheobronchial surface clean (Table 1).

This mucus production is usually not apparent, as it is mainly swallowed during the night [7,8]. In a healthy person, pulmonary mucus is composed of ~97% water, 0.9% salt, and the remaining ~2% a mixture of macromolecules [7,8,9]. Among the latter, in addition to antimicrobial proteins and peptides, which include lysozyme, lactoferrin, and defensins, there are glycoproteins, also known as mucins, which give mucus its gel-like consistency and help trap particles and microorganisms. At least 22 different mucins have been identified in humans, designated MUC1 through MUC22. They are classified based on their structure and cellular localization into two main categories: secreted mucins, which are released into the extracellular space and are primarily responsible for forming mucus gels, and membrane-bound mucins, which remain attached to the cell surface and play roles in cell signaling, adhesion, and protection [10].

In pathological conditions, mucus accumulation represents a classic clinical problem in the chronic and exacerbating phases of many airway diseases. Recent translational data suggest that muco-obstructive lung diseases are typically associated with hyperconcentrated, i.e., dehydrated, mucus [6]. Basic science studies have contributed two major insights into the mechanisms that mediate intrapulmonary mucus accumulation. First, electrophysiological studies suggest that imbalances in Na^+^/fluid absorption versus Cl^−^/fluid secretion cause increased mucus concentrations (Table 1). Second, polymer physics studies suggest that mucus hyperconcentration generates mucin polymer-dependent osmotic forces that compress the mucus layer onto airway surfaces, ultimately producing the mucus accumulation, particularly in small airways, that characterizes muco-obstructive lung diseases [10,11]. Therapies for muco-obstructive lung diseases can be directed toward the upstream ion transport defects that produce hyperconcentrated mucus, as demonstrated by the highly effective modulators that restore CF Transmembrane Conductance Regulator (CFTR) function, and/or therapies designed to broadly rehydrate airway surfaces, including inhaled osmolytes. Therapeutic needs for the future include more durable/effective hydrating agents and agents that break up accumulated intrapulmonary mucus, i.e., effective mucolytics. In Table 1, we provide a comparative overview of the main features distinguishing physiological airway mucus from pathological mucus, including the composition, biophysical state, regulatory mechanisms, and clinical manifestations.

Mucins [9] are the central macromolecular components of airway mucus, and they are crucial for several reasons: (a) mucins represent the structural framework of mucus, (b) they provide a steric and electrostatic barrier with a protective role against pathogens, (c) by determining mucus viscosity and elasticity, mucins regulate mucus clearance and directly affect mucociliary transport, and (d) membrane-bound mucins (e.g., MUC1, MUC4, MUC16) are involved in cell signaling, adhesion, and epithelial repair, exerting a dynamic defense system. They balance protection, hydration, and clearance functions. In summary, mucins are essential “scaffold molecules” that make airway mucus a physical barrier that is disrupted when mucin production, structure, or concentration becomes abnormal (see Appendix A for multiscale structure of mucus).

**Figure 1:**
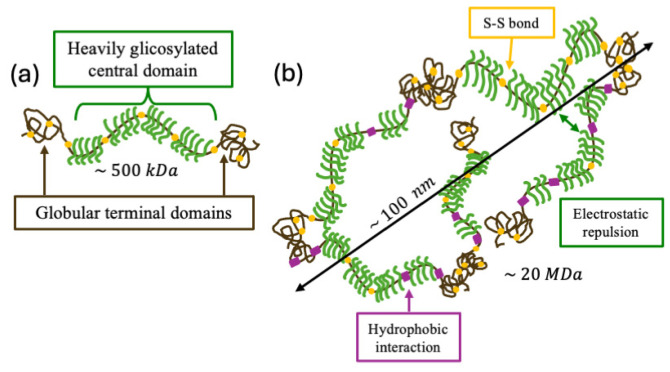
Structural organization of mucins: (**a**) schematic of a single mucin macromonomer with glycosylated central domain and non-glycosylated terminal regions; (**b**) polymer assembly through intermolecular disulfide bonds forming the mucus network.

Figure 1 shows a simplified schematic of the structural organization of mucins.

As shown in Figure 1a, each mucin unit (referred to as a macromonomer due to its substantial molecular weight of ~500 kDa) [11,12] consists of a heavily glycosylated central domain flanked by two terminal regions that are globular and devoid of glycosylation. These macromonomers are covalently linked via intermolecular disulfide bonds, forming linear polymers with molecular weights reaching tens of megadaltons (see Figure 1b) [13]. The cysteine residues responsible for this multimerization are predominantly located in the non-glycosylated terminal domains, where they also form intramolecular disulfide bridges that stabilize the globular structure. In addition to disulfide bonding, salt bridges are reported to contribute to the stabilization of these terminal regions [14,15]. These are specific electrostatic interactions between oppositely charged residues (e.g., lysine and glutamate) or between negatively charged domains of mucins and DNA, which are stabilized by divalent cations [14].

Mucins possess both hydrophilic glycosylated domains in the central region and hydrophobic globular domains in the terminal regions. This dual functionality allows mucins to interact with a wide range of proteins and nucleic acids, giving mucus its characteristic biophysical properties. Under normal conditions (i.e., physiological pH and salt concentration), mucin chains interact mainly with repulsive and attractive forces. The repulsive forces are essentially those due to the negative charges present in the glycosylated regions, while the attractive forces are due to hydrophobic interactions between residues present in the globular regions [14]. Far from being an ideal homogeneous network, mucus also presents morphologic structures, such as filaments and threads, made up of bundles of mucin chains held together by calcium-mediated bonds. These morphologic structures reflect the formation of mucins within the goblet cells and tend to evolve over time following their secretion into mucus [16].

All these structures and interactions contribute to the stability and integrity of the three-dimensional network formed by mucins, giving mucus its peculiar viscoelastoplastic characteristics (see Appendix A) [17]. In a micro- and macro-rheological study, Wagner et al. investigated how these properties can be significantly modulated by environmental and chemical–physical variables. Specifically, they studied how changes in pH, ionic strength, and presence of surfactants alter the complex equilibrium within mucus [14]. The effect of pH has been linked to variations in the non-glycosylated regions. At neutral pH, mucins have a negative charge due to the deprotonation of the carboxylic groups present in the amino acid residues. This charge causes electrostatic repulsion between the various parts of the molecule, inducing globular conformation and allowing mucins to be extended and flexible [15]. This conformation is in part the result of the salt bridges mentioned previously. In fact, the presence at neutral pH of both negative and positive charges (the latter being due to the protonation of basic amino acids, such as lysine) determines the formation of salt bridges. This guarantees the structural stabilization of the mucin by keeping the hydrophobic regions, present in the flexible globular domains, folded towards the inside of the globule and, therefore, not accessible [18]. Following acidification, a loss of flexibility of the mucus structure occurs due to the protonation of the carboxylic groups of the non-glycosylated amino acid chains, leading to the destruction of the salt bridges and the consequent unfolding of the mucin chains. This opening provides access to the previously hidden hydrophobic regions that can then form new bonds, allowing a mesoscopic phase separation of the mucus into mucin-rich domains, held together by hydrophobic bonds and reduced electrostatic repulsion. These molecular rearrangements at low pH lead to an increase in rigidity of the mucus gel, as observed macroscopically by measuring the rheological moduli: pH decreases drive meso-phase separation in mucus. The analysis of the microstructure by particle tracking confirmed the presence of mucus heterogeneity due to the identification of two populations of particles, a group of slow particles trapped within mucin-rich domains and a second group of fast particles capable of diffusion [14].

Variation in physical properties has also been observed by changing the ionic strength. The increase in salt concentration determines a reduction in the electrostatic repulsion between the glycosylated chains, reinforcing the interactions between the mucin chains. This can be observed macroscopically by an increase in the elastic modulus and microscopically by a decrease in the mobility of the test particles. The combined methods at different scales confirm the stiffening of the structure and the presence of a single homogeneous phase (constant heterogeneity index) as the ionic strength increases.

Disulfide-reducing agents, such as dithiothreitol (DTT) and N-acetylcysteine (NAC), alter the physical properties of mucus by targeting the disulfide bonds that “stabilize” the mucin polymer network. When these bonds are reduced, there is a significant decrease in the viscosity and elasticity of the mucus, which has been linked to a change in mucin interactions, rather than to a reduction in the molecular weight of the mucins, as initially hypothesized [19,20]. It is worth noting that although disulfide reduction facilitates the clearance of mucus from the airways, excessive disruption of mucin structure may compromise the protective function of the mucus barrier, potentially increasing vulnerability to pathogens [21].

Surfactants can bind to the hydrophobic regions of mucins, thereby inhibiting the hydrophobic interactions between their non-glycosylated domains. This disruption weakens the structural integrity of the mucus, leading to decreased rigidity and overall stability. Experimentally, this effect is reflected in a decline in the macro-rheological elastic modulus as the surfactant concentration increases. Concurrently, micro-rheological analyses reveal a reduction in mucus heterogeneity, evidenced by the emergence of a single population of particles exhibiting Brownian motion, indicative of a more uniform, homogeneous phase [14].

Other variations in mucin interactions may occur in pathological mucus. In patients affected by cystic fibrosis, for instance, the concentration of mucins can reach up to 6% in mass [22]. This, in turn, leads to an increase in the viscoelastoplasticity of the mucus, which impairs the mucociliary clearance system, causing chronic inflammation of the airway. This is confirmed by the analysis of the microstructure of pathological mucus through micro-rheological techniques (see Appendix A). Particle tracking measurements demonstrate that the mean square displacement is a function of the concentration of mucins [22]. It is clear that changes in mucus concentration have a more pronounced impact on its rigidity than the stiffening effects caused by pH alterations [8].

Another example of pathological variation occurs in the presence of inflammation. Under this condition, neutrophils release a greater quantity of DNA, increasing its concentration in the mucus [23,24]. Due to their negative charges, both DNA and mucins can interact through salt bridges facilitated by divalent cations such as Ca^2+^ or Mg^2+^, promoting cross-linking and enhancing the viscoelastic properties of mucus [2]. In fact, the increase in DNA concentration compared to that of mucins determined an increase in the viscosity and elasticity of the mucus and a reduction in mucociliary transport, as demonstrated by micro-rheological tests (see Appendix A) [25,26]. The increase in the concentration of free radicals in pathological mucus determined an increase in the rigidity of the mucus and a reduction in mucociliary transport due to the formation of new covalent bonds in the mucin network and the increase in molecular weight [27]. A possible modification of the interaction of trefoil factors (TFFs) with mucins has been found to be decisive for the mechanical properties of mucus [28]. In fact, a variation in the interaction between TFF3 and the glycosylated domains of mucins determined an increase in the elastic modulus, the viscous modulus and the viscosity of the mucin solution [29].

**Figure 2:**
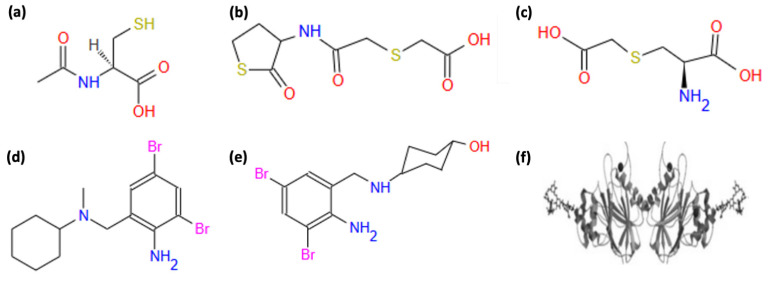
Chemical structures of selected mucoactive agents (including true mucolytics and non-cleaving agents). (**a**) N-acetylcysteine: association of an acetyl group and a cysteine group (C5H9NO3S). (**b**) Erdosteine: thiol derivative (C8H11NO4S2). (**c**) Carbocisteine: cysteine derivative (C5H9NO4S). (**d**) Bromhexine: alkaloid derivative (C14H20Br2N2). (**e**) Ambroxol: bromhexine metabolite (C13H18Br2N2O). (**f**) Dornase alfa (Pulmozyme^®^): peptide mucolytic, 260 amino acids.

In the context of the complex intermolecular interactions outlined above, the clinical use of mucolytics at the clinical level has encountered non-linear efficacy. Mucolytics exert many different actions, besides those leading to the clearance of pathological mucus, i.e., sputum. Those discussed in this review are represented in Figure 2.

The aim of this review is to critically present the variety of in vitro, ex vivo and in-patient effects of mucoactive agents in the treatment of muco-obstructive lung diseases, namely COPD and CF, and bronchiectasis that can derive from CF and COPD but is not always associated with these conditions [30]. We will mainly focus on COPD and CF, since they share pathophysiological characteristics. CF is an autosomal recessive disease caused by mutations in the *CFTR* gene, leading to impaired ion and fluid homeostasis at the apical surface of airway epithelial cells. This, in turn, leads to a marked reduction in mucociliary clearance, eventually promoting chronic infection and inflammation [31]. COPD is a heterogeneous condition traditionally linked to long-term exposure to toxic particles and gases, particularly tobacco smoke, leading to chronic airway inflammation, mucus hypersecretion, and impaired gas exchange [32]. More recently, acquired CFTR dysfunction [33,34] has been implicated in COPD pathogenesis, especially in chronic bronchitis, which shares with CF goblet cell metaplasia and mucin hypersecretion, mucus obstruction of the small airways and chronic bacterial infection [35,36,37]. Components of tobacco smoke, including acrolein and cadmium, impair both the expression and function of the CFTR protein. This occurs through reduced mRNA and protein levels, accelerated endocytosis, and a lower probability of channel opening [38,39]. Specifically, acrolein—an aldehyde found in smoke—can directly modify CFTR’s amino acid residues [40]. Environmental factors like arsenic exposure also contribute to CFTR dysfunction and bronchitis symptoms [41]. Additionally, pollutants such as cigarette smoke and cadmium upregulate microRNAs like miR-101 [42], which suppress CFTR expression [43]. Proteases like neutrophil elastase, released during chronic bronchitis inflammation, further degrade the CFTR protein while increasing ENaC (epithelial sodium channel) expression [44,45,46]. This synergy worsens airway surface liquid (ASL) dehydration. Since CF and COPD (particularly chronic bronchitis) share these pathological mechanisms, CFTR modulators—currently used for personalized CF treatment—are now being investigated as potential therapies for COPD [34]. The neutrophil-dominated immune response elicited in both COPD and CF results in tissue damage and permanent enlargement of bronchi/bronchioles, i.e., bronchiectasis, with further mucus accumulation and a vicious cycle of infection and inflammation, which worsens the clinical trajectory of these patients.

A unifying mechanistic model for muco-obstructive diseases (such as CF and COPD) describes a self-perpetuating pathological feedback loop where oxidative stress transforms mucus from a transportable fluid into a stagnant, elastic solid. In muco-obstructive diseases, there are several sources of oxidative stress, both endogenous and exogenous, including CFTR deficiency [47,48]. These oxidants drive the formation of inter-chain disulfide bonds between cysteine-rich domains of mucins, creating a rigid, covalently cross-linked mesh rather than a loosely associated gel [27]. This increases the elastic modulus (G′), making the mucus highly resistant to ciliary clearance and cough-driven transport. Extracellular DNA (eDNA), primarily derived from neutrophil extracellular traps (NETs), acts as a secondary structural component, since it physically intertwines with the mucin fibers. Moreover, the negative charge of the DNA backbone can interact with mucin proteins, further densifying the “mesh” and reducing pore size [27]. NETs significantly increase mucus viscoelasticity (macro-rheology) and significantly decrease mesh pore size of the mucus and movement of muco-inert nanoparticles through the mucus (micro-rheology) [49]. This traps bacteria and inflammatory mediators more effectively. Thus, an inflammatory “vicious cycle” ensues [33]. Stagnant mucus provides a niche for bacterial colonization. Bacteria and their products further stimulate neutrophil recruitment. Massive neutrophil influx leads to high levels of neutrophil elastase (NE), which damages the epithelium and triggers more mucin secretion (hypersecretion) [50]. Chronic inflammation and reactive oxygen species (ROS) impair CFTR function (even in non-CF patients), leading to ASL dehydration [51,52,53], which concentrates the cross-linked mucins and eDNA even further.

This model may explain why traditional hydration (saline) [54] or targeting DNA alone (DNase) [55] often fails to fully clear the airways. Breaking the covalent disulfide bonds with thiol-reducing agents (like NAC or newer, more potent reducers) [27] is often necessary to decouple the mucin network.

Based on these considerations, we have thus considered in this review these two pathological entities as correlated and analyzed the role of mucoactive agents also in relation to bronchiectasis.

In particular, we examine mucoactive agents according to four complementary axes: (i) their predominant biological target within the mucus compartment, including disulfide-rich mucin networks, extracellular DNA burden, mucin glycosylation, airway hydration, and mucociliary transport; (ii) the strength and limitations of the available clinical evidence, taking into account study design, treatment duration, endpoint selection, and population heterogeneity; (iii) disease- and phenotype-specific applicability; and (iv) the implications for precision airway medicine. Within this framework, the key issue is not whether mucolytics “work” in general but rather in which biological and clinical context, at which dose, and for which outcome they are most likely to provide meaningful benefit.

## 2. Methods: Literature Search and Selection Criteria

We conducted a narrative review with a structured search of PubMed/MEDLINE and Scopus from January 2000 to October 2025 using controlled vocabulary and free-text terms for mucolytic agents and muco-obstructive lung diseases (COPD, bronchiectasis, cystic fibrosis). Inclusion criteria: randomized or observational studies reporting clinical or physiological outcomes (exacerbations, lung function, symptoms, quality of life). Exclusion criteria: case reports, non-systematic narrative pieces without primary data, and non-English full texts. We screened titles/abstracts and assessed full texts for relevance, prioritizing recent high-quality evidence (meta-analyses, RCTs).

For the purpose of critical reappraisal, the retrieved evidence was qualitatively appraised according to study design (randomized controlled trials, observational studies, post hoc analyses, and meta-analyses), sample size, treatment duration, endpoint hierarchy (symptom relief and sputum properties versus exacerbations, lung function, and quality of life), and consistency across studies. Particular attention was paid to clinically relevant sources of heterogeneity, including dose, route of administration, background therapy, exacerbation history, chronic bronchitis predominance, infection status, and disease-specific mucus biology. Whenever possible, the interpretation of clinical outcomes was integrated with mechanistic considerations in order to identify settings in which a given mucolytic may be biologically plausible yet clinically diluted by unselected trial populations.

## 3. Drug Delivery and Airway Surface Layer

Inhalation represents one of the most promising therapeutic strategies for drug delivery to lungs in relation to very important pathologies such as chronic respiratory diseases (i.e., asthma, COPD and CF) [56,57] and lung cancer [58]. Indeed, the advantages of pulmonary delivery over systemic delivery include the rapid degradation of therapeutics, the capability to bypass systemic first-pass metabolism, smaller doses of therapeutics to get the desired effects, the limitation of non-specific toxicity, the possibility of targeting poorly reachable lungs regions such as distal bronchi [59], and the possibility of delivering traditional drugs (antibiotics, bronchodilators, anti-inflammatory, osmotic agents, mucolytics), gene therapy drugs (DNA, RNA, si-RNA, and so on), peptides, proteins [60], and plant-based drugs [61]. Nevertheless, drug delivery to lungs must account for the presence of the defensive barrier represented by the ASL [62,63]. The ASL is constituted by the upper mucus layer (ML), mainly composed of a mucin network, and the lower periciliary layer (PCL), hosting membrane-spanning mucins and large mucopolysaccharides that are tethered to cilia, microvilli, and the epithelial surface [51]. Consequently, regardless of the different delivery systems that can be considered [64], a successful therapeutic approach implies drugs crossing the ASL, i.e., drug diffusion through the ASL nanostructure. This aspect plays a relevant role if we consider that in CF and COPD patients, mucus concentration and its three-dimensional organization are considerably altered. Indeed, the solid content of mucus increases from a normal value of approximately 2% to as high as 8% [65], while mesh size decreases from 300–800 nm in healthy subjects [66] to 60–300 nm in CF patients [67]. Thus, the determination of mucus nanostructure is not only important per se (as it is connected to disease severity) but it is fundamental for drug delivery as drug transport inside the ASL is affected by possible drug–polymer interactions and by mesh size distribution. If traditional drug dimensions span between 1 and 10 nm [68], in many situations, drugs must be bound to a carrier aimed at protecting them from different environmental factors such as enzymes. The dimensions of these carriers can span from 20–50 nm (gold nano-stars and silver nanoparticles) [69] to 100–200 nm (liposomes) [70] and to 300–600 nm (lipid particles). In this framework, rheology and, more recently, LF-NMR [71], when used together, have proven to provide a precise picture of mucus nanostructure. Indeed, while rheology provides the average mesh size, LF-NMR allows determination of how bigger and smaller meshes are distributed around the average mesh (see Appendix A). Thus, the contemporaneous use of these techniques gives rise to a synergistic approach enabling information to be obtained about drug diffusion inside the ASL and about the nanostructure alterations induced by the administration of drugs such as mucolytics [72]. Clearly, this strategy helps a lot in pursuing the modern concept of a patient-centered approach [73].

## 4. N-acetylcysteine

### 4.1. Mechanisms of Action of N-acetylcysteine

Discovered in the early 1960s for treating chronic lung disease by reducing sputum viscoelasticity [74], N-acetylcysteine (NAC) is a mucolytic agent whose therapeutic effects stem from its intrinsic reducing power. This property enables NAC to cleave disulfide bonds within mucus, thereby making the mucus more fluid-like. Additionally, NAC’s reducing activity contributes to the inhibition of mucus secretion, cellular hyperplasia, and MUC5AC expression [75].

Beyond its mucolytic role, NAC’s reducing capacity underlies its antioxidant, anti-inflammatory, and immunomodulatory effects [76,77,78,79,80]. As a thiol-containing molecule, NAC acts directly as a scavenger of reactive oxygen species (ROS) [81] and indirectly by replenishing intracellular glutathione (GSH), a key antioxidant. This dual antioxidant mechanism is facilitated by NAC’s ability to donate cysteine, the rate-limiting precursor in GSH synthesis, and to activate nuclear factor erythroid 2-related factor 2 (Nrf2), a transcription factor regulating antioxidant and cytoprotective enzymes. In COPD, oxidative stress plays a central role in pathogenesis [82]. NAC, through its reducing action, enhances GSH synthesis and directly scavenges ROS like hydrogen peroxide, mitigating cellular oxidative damage. This redox modulation attenuates the activation of nuclear factor kappa-light-chain-enhancer of activated B cells (NF-κB), a master regulator of inflammation, and downstream cytokine production (e.g., interleukin (IL)-6), which are elevated during COPD exacerbations [80,83].

The anti-inflammatory effects of NAC also originate from its redox-modulating activity. By suppressing redox-sensitive pathways such as NF-κB and mitogen-activated protein kinase (MAPK), NAC reduces the expression of pro-inflammatory mediators including cyclooxygenase (COX)-2, matrix metalloproteinase (MMP)-3/4, and intercellular adhesion molecule (ICAM)-1 [80]. Experimental models further support NAC’s redox-based mechanisms. In ex vivo models of acute COPD exacerbation, NAC reduced oxidative markers (H_2_O_2_, malondialdehyde, nitric oxide) and inflammatory cytokines (IL-6), implicating neurokinin receptor pathways [84,85]. In viral infection models using synthetic dsRNA (Poly I:C), NAC pre-treatment prevented ROS accumulation and restored thiol levels while suppressing NF-κB activation and IL-6/IL-8 expression [86]. However, in the βENaC-overexpressing mouse model of cystic fibrosis, NAC failed to reduce mucus plugs, likely due to its limited mucolytic potency and short residence time, highlighting the need for more potent reducing agents [87].

Overall, NAC’s therapeutic effects are dose-dependent and rooted in its reducing nature: (i) low doses (≈200 mg/day) primarily reduce neurokinin A and IL-6, and (ii) high doses (≥1200 mg/day) more effectively suppress NF-κB and pro-inflammatory cytokines (IL-1β, tumor necrosis factor (TNF)-α, IL-8), sustaining antioxidant and anti-inflammatory activity [80,88].

### 4.2. Administration Route and Clinical Effects of N-acetylcysteine

The administration of NAC can be performed orally, intravenously and by inhalation. Each of these methods is selected based on the specific needs of patients and the nature of their disease. The oral route of administration of NAC is widely utilized on account of its convenience and effectiveness in reducing exacerbations and improving symptoms in patients suffering from COPD and chronic bronchitis [83] In particular, there is evidence that high levels of NAC (>1200 mg/die) are associated with reduction in the risk of COPD exacerbations, and normal levels (600 mg/die) are associated with symptom improvement in chronic bronchitis [89]. In a randomized controlled trial investigating the efficacy and safety of long-term treatment with high-dose NAC (600 mg, twice daily) in patients with COPD of GOLD stage 1–2, the treatment neither reduced the annual rate of total exacerbations nor improved lung function [90]. However, it significantly lowered the annual rate of moderate or severe exacerbations compared to the placebo and was very well tolerated.

Intravenous (IV) NAC is used in more acute settings, such as in patients with respiratory diseases characterized by abnormal mucus secretion while seriously ill [91]. The use of this route is particularly recommended for patients who are hospitalized or for whom oral administration is not a viable option. In a large, multicenter, randomized, controlled trial on patients with acute bronchitis, chronic bronchitis and exacerbations, emphysema, CF, and bronchiectasis, IV NAC showed consistent and statistically significant superiority over the placebo and non-inferiority to ambroxol in improving sputum viscosity and expectoration difficulty after 7 days of treatment [91]. Despite the presence of a limited number of studies examining the impact of nebulized NAC administration, the hypothesis that inhalation could serve as a delivery route for NAC in pathological conditions where direct action on the airways is advantageous warrants further exploration. This method may facilitate targeted delivery to the respiratory tract, increasing its concentration in situ and enhancing the mucolytic effect. In a study by App et el. with aerosolized NAC, rheological analysis of sputum showed a dose-dependent decrease in sputum viscoelasticity, accompanied by a decrease in sputum solids content and an increase in chloride and sodium concentrations [92]. However, neither sputum neutrophil elastase nor bronchial secretory inhibitor changed significantly following NAC administration. Conversely, a single aerosolization of NAC in a small group of patients (*n* = 5) did not produce mucus reduction, likely due to the limited residence time of NAC in the airways [87]. In 2024, a prospective, single-arm, open-label, phase IV, multicenter trial was conducted in 100 enrolled COPD patients to establish the long-term effectiveness of nebulized NAC. The phlegm (more than normal amount of thick mucus) score at 12 weeks decreased significantly in comparison to the baseline. This significant reduction was also observed at 4 and 8 weeks of treatment with nebulized NAC. Moreover, the treatment was well tolerated, with good overall compliance and no serious adverse events [93]. No relevant evidence regarding the comparative clinical effectiveness of nebulized NAC versus oral NAC for patients requiring mucous secretion clearance was identified [94].

Regarding the adverse effects of NAC, they can range from mild to severe and largely depend on the formulation and dosage used. However, an extensive review of multicentric medical records has shown that both intravenous and oral NAC are generally associated with minimal side effects [95]. The most commonly reported side effects of oral NAC are vomiting and diarrhea, with their incidence increasing by 50% and 43.5%, respectively, after 16 to 18 days of use [96]. Other less frequent side effects, occurring in fewer than 5% of cases, include increased blood pressure, respiratory distress, chest pain, fever, rectal bleeding, headache, hypotension, lethargy, and skin allergies. Aerosolized NAC is often poorly tolerated because of its irritant properties and strong, unpleasant odor that resembles rotten eggs (due to sulfur), which may trigger vomiting. Intravenous administration of NAC can also cause symptoms such as nausea and vomiting, with a frequency of up to 9% and a higher risk of anaphylactic reactions [97].

### 4.3. Immunomodulatory and Antioxidant Effects of N-acetylcysteine in Patients

A meta-analysis stated that mucolytics reduce exacerbations and hospitalizations in patients with stable COPD and have a safety profile comparable to that of a placebo [98], but few clinical studies have analyzed anti-inflammatory and antioxidant effects of NAC in COPD and bronchiectasis [99]. A small pilot study observed that high-dose NAC treatment (1800 mg daily for 3 months) caused a significant correlation between image-based airway resistance values and glutathione levels after treatment and with glutathione peroxidase at baseline; this indicates that NAC can activate antioxidant properties, eventually resulting in a reduction in airway resistance [100]. A phase IV clinical trial (ClinicalTrials.gov NCT00969904), investigating the effect of high-dose NAC on small airway resistance and oxidative stress and inflammation, has been completed. Finally, a randomized, double-blind, placebo-controlled study in patients with bronchiectasis evaluated 2400 mg/day oral NAC for 6 weeks, finding that sputum neutrophil elastase fell by 47% and MUC5B increased by 48% in the NAC group relative to the placebo [101]. As far as the MUC5B results are concerned, it can be speculated that its increase may reflect the breakage of the mucin disulfide bonds by NAC, resulting in the thinning of the sputum and thus allowing easier expectoration, a hypothesis that seems to be confirmed by the rheological and LF-NMR characterization of mucus before and after NAC administration in in vitro tests (see Appendix A for details).

Short-term high oral doses of NAC (up to 1.0 g per day, three times daily, for 4 weeks) efficiently targeted circulating neutrophils without improving pulmonary functional tests in CF patients with stable disease. In particular, NAC treatment significantly increased GSH levels in CF blood neutrophils and decreased the neutrophil count in CF airways, as well as IL-8 sputum and elastase levels [102]. In a CF phase II trial of 12-week therapy with oral low-dose (700 mg/daily) and high-dose (2800 mg/daily) NAC, although these treatments did not alter clinical parameters or inflammatory markers, such as sputum IL-8 and TNF-α, extracellular GSH in induced sputum tended to increase with high-dose NAC [103]. A study which aimed to investigate the effect of high-dose, orally administered NAC (1200 mg × 2/day for 30 days) on oxidative stress markers found that the treatment of CF patients significantly decreased the plasma level of the oxidized form of ascorbic acid and increased the level of vitamin C, while a non-statistically significant improvement in lung function was observed [104]. In a long-term study (24 weeks) of orally administered NAC, treated CF patients maintained their lung function, while that of placebo recipients declined; however, no effect on sputum neutrophil elastase activity and other selected biomarkers of inflammation (sputum neutrophil count, IL-8 either sputum or plasma) or oxidation status (GSH in whole blood) was detected [105].

Based on the overall survey of these studies, it is envisioned that trials involving larger patient cohorts and favoring oral (rather than aerosolized) administration of NAC may better demonstrate its antioxidant and anti-inflammatory efficacy in CF. The sulfhydryl group of NAC may interact with ROS present in the airways of CF subjects, potentially exacerbating oxidative stress over the long term [106]. Therefore, future trials should aim to define the optimal therapeutic window and dosing regimen that can maximize NAC’s systemic antioxidant effects while minimizing the risk of local pro-oxidant activity within the airway microenvironment of CF patients.

Interestingly, we verified that NAC’s action on the polymeric network permeating CF sputum can be detected by means of LF-NMR (see Appendix A). Indeed, its addition in powder form to two sputum samples (NAC mass fraction concentration ω_1_ = 0.25 and ω_2_ = 0.21; *T*_2m−1_ = 1652 ms; *T*_2m−2_ = 1145 ms) implied a *T*_2m_ increase already after 30 min incubation at 37 °C (*T*_2m−1_ = 1916 ms; *T*_2m−2_ = 1619 ms). As this increase was equal to 16% and 41%, respectively, of the original *T*_2m_ (untreated sample), we should conclude that NAC’s action (reduction in polymeric network connectivity, i.e., increase in the average mesh size) was, reasonably, more evident for sample N2, corresponding to worse clinical conditions (lower *T*_2m_). For both samples, *T*_2m_ underwent a small reduction (*T*_2m−1_ = 1856 ms; *T*_2m−2_ = 1512 ms) after 24 h following NAC addition (incubation at 37 °C), implying a slight tendency of the polymer network towards restructuring. Notably, the addition of NAC (ω = 0.17) to another aliquot of sample N1 implied, after 30 min at 37 °C, a shift from gel-like rheological behavior (*G*′ > *G*″) to solution behavior (*G*′ < *G*″). This, again, can be reasonably attributed to a lack of connectivity among the polymeric network due to NAC’s action. Notably, these findings are in line with what was found by Suk and colleagues, who studied NAC’s effect on the sputum of CF patients [107]. Indeed, using particle tracking experiments (PEG-coated nanoparticles) in NAC-treated sputum, they found an increase in the average mesh size from 145 ± 50 nm to 230 ± 50 nm upon NAC treatment.

### 4.4. Critical Appraisal and Translational Positioning of N-acetylcysteine

The clinical signal for NAC in COPD should be interpreted with caution because the label “COPD” encompasses biologically heterogeneous mucus phenotypes. Apparent benefits in exacerbation reduction are more coherent in studies using higher doses and longer treatment durations but are likely diluted by variability in chronic bronchitis burden, baseline exacerbation risk, inhaled background therapy, and endpoint definition. Moreover, not all studies have enrolled populations enriched for productive cough or chronic mucus hypersecretion, which may partly explain the inconsistent magnitude of benefit across trials. Therefore, NAC should not be viewed as a uniformly effective therapy across the entire COPD spectrum but rather as a candidate adjunctive treatment in selected chronic bronchitic or frequent-exacerbator phenotypes, in which mucus hyperconcentration and oxidative stress are more likely to coexist. This interpretation also helps explain why mechanistic plausibility has often exceeded the strength of the clinical effect observed in unselected populations.

## 5. Erdosteine

### 5.1. Mechanisms of Action of Erdosteine

Erdosteine [N-(carboxymethylthioacetyl)-homocysteine thiolactone] is a thiol-based prodrug that belongs to the same chemical family as NAC, yet it differs in its mechanism of activation and redox behavior. While NAC is pharmacologically active in its native form due to its free thiol group, erdosteine requires metabolic activation to release its active metabolite, known as Metabolite 1 (M1), which contains a free thiol group. This thiol group is responsible for the mucolytic activity of M1, enabling it to cleave disulfide bonds in mucus, thereby reducing its viscoelasticity, similar to NAC’s direct mucolytic mechanism [108]. However, unlike NAC, which acts immediately as a reducing agent, erdosteine’s activity is delayed and dependent on enzymatic conversion, making it a prodrug with a more controlled release of thiol functionality. This distinction is important in terms of pharmacokinetics and tissue targeting. In addition to its mucolytic properties, the active metabolite of erdosteine exhibits antioxidant activity by directly scavenging ROS, a function it shares with NAC [109]. However, while NAC also replenishes intracellular GSH by serving as a cysteine donor, erdosteine does not significantly contribute to GSH synthesis, and its antioxidant effect is primarily due to direct radical scavenging via the thiol group of M1 [84]. Erdosteine also demonstrates anti-inflammatory properties, which, like NAC, are mediated through the inhibition of redox-sensitive signaling pathways such as NF-κB. However, the extent and mechanism of this inhibition may differ due to differences in cellular uptake, metabolic activation, and redox potential [84,110].

In summary, although both erdosteine and NAC belong to the thiol-based class of mucolytics and share some overlapping biochemical targets, their mechanisms of action are not identical. NAC is an immediate-acting thiol donor with both mucolytic and glutathione-replenishing properties, while erdosteine is a prodrug that must be metabolized to release its active thiol, which then exerts mucolytic and antioxidant effects primarily through direct ROS scavenging rather than GSH synthesis.

### 5.2. Administration Route and Clinical Effects of Erdosteine

Erdosteine is administered orally. Results from initial clinical studies have indicated that erdosteine is an effective and well-tolerated treatment in patients with chronic obstructive bronchial disease, with efficacy at least equivalent to that of ambroxol in reducing symptoms [111]. One of these trials was conducted to evaluate the efficacy and safety of erdosteine in the treatment of chronic bronchitis during an infective exacerbation phase. The active group was treated with erdosteine (300 mg twice daily) and the control group with a placebo in association with amoxycillin (1500 mg daily) for a maximum period of 10 days. The analysis demonstrated a more rapid improvement in breathlessness, cough and sputum viscosity in patients who received the combination of amoxicillin and erdosteine compared with those who received the antibiotic alone [112]. In 1999, Aubier et al. conducted a multicenter, double-blind, randomized, parallel-group study to evaluate the efficacy and tolerance of erdosteine treatment compared to a placebo in chronic bronchitis patients (*n* = 170). The global index of efficacy, which considers the frequency, severity, and intensity of the cough, as well as dyspnea, was found to be statistically lower in patients treated with erdosteine compared to those treated with a placebo [113].

The exacerbation rate in COPD patients can be reduced by drugs with antioxidant, anti-inflammatory and mucolytic properties, such as NAC [114] and CCis [115]. However, little is known about the efficacy of these drugs in reducing the duration of exacerbation. The RESTORE study was conducted with the objective of demonstrating the effectiveness of erdosteine in reducing the rate and duration of COPD exacerbations in patients with Global Initiative for Chronic Obstructive Lung Disease stage II/III. Patients received erdosteine 300 mg twice daily or a placebo in addition to their usual COPD therapy for a period of 12 months. Erdosteine significantly reduced the rate of mild exacerbations in the intention-to-treat population of 445 patients, in comparison with the placebo group. No significant difference was observed in the rate of moderate and severe exacerbations. Furthermore, erdosteine was demonstrated to reduce the duration of exacerbation, irrespective of the severity of the event. The study demonstrated that erdosteine led to a substantial improvement in both subject and physician subjective severity scores while concurrently reducing the utilization of reliever medication [116]. A post hoc study was carried out to understand whether erdosteine could have the same effects in patients with moderate COPD [117]. Indeed, also in this subgroup of patients, adding erdosteine to conventional therapy resulted in fewer exacerbations and a shorter duration of exacerbations than in the placebo group. In 2019, an interesting study was conducted to compare the efficacy of erdosteine (600 mg/die), carbocisteine (1500 mg/die) and NAC (1200 mg/die). This analysis found that erdosteine was the only one to reduce the risk of hospitalization for acute exacerbation of COPD and the risk of developing at least one acute exacerbation [118].

While erdosteine lacks extensive investigation within the context of CF [119], many standard treatments for bronchiectasis are generally adapted from the clinical evidence and experience established in CF care [30]. A short-term small (*n* = 30) RCT in elderly patients with radiographic bronchiectasis and chronic hypersecretion found a reduction in mucus density and purulence, a significant improvement in measured forced lung volumes and improved clinical outcomes (better cough and dyspnea scores, 6 min walk test) on day 15 with erdosteine 225 mg BID [120]. To estimate the beneficial effect of erdosteine on bronchiectasis in the long term, an international multicenter, double-blind, placebo RCT aimed to assess whether 12 months of erdosteine had an effect in children and adults with non-CF bronchiectasis, in particular on the rate of exacerbations [121]. However, results are still awaited. In conclusion, further high-level evidence of erdosteine efficacy in CF or bronchiectasis is warranted.

Erdosteine is associated with a low incidence of adverse events, the majority of which are mild and primarily gastrointestinal in nature [111]. This favorable safety profile makes it a well-tolerated therapeutic option. In a study by Yildirim et al. [122], treatment with erdosteine at a dosage of 10 mg/kg/day for five days was shown to effectively prevent oxidative injury induced by cisplatin in the kidney. These findings suggest that erdosteine not only has a good safety margin but also possesses protective antioxidant properties that may mitigate cisplatin-related nephrotoxicity.

### 5.3. Antioxidant and Anti-Inflammatory Effects of Erdosteine

In vitro studies have revealed the antioxidant activity of erdosteine and its metabolite M1 at the level of human and rat neutrophils [123,124], and there is evidence from animal models that erdosteine protects against various types of tissue injuries mediated by products of oxidative stress [125]. As to erdosteine’s anti-inflammatory effects, it has been shown to inhibit TNF-α, IL-1β and free radical production in rat alveolar macrophages [126] and lipopolysaccharide (LPS)-induced NF-κB activation in mouse macrophages [127]. Hayashi et al. reported that erdosteine inhibited the LPS-induced neutrophil influx in a mouse lung injury model, although it did not affect the increased level of TNF-α in the bronchoalveolar lavage (BAL) fluid [128]. Interestingly, post-treatment of rats with erdosteine and NAC significantly reduced the rate of LPS-induced epithelial cell apoptosis as well as increases in the local production of TNF-α and vascular endothelial growth factor (VEGF) and epithelial myeloperoxidase (MPO) activity. Erdosteine was demonstrated to be superior to NAC in these effects [129]. Erdosteine can also inhibit the chemotactic peptide N-Formylmethionine-leucyl-phenylalanine (fMLP)-induced elastase release of neutrophils in a concentration-dependent manner at concentrations that are achievable in the clinical setting [130]. In summary, the observed inhibition of the release of elastase and reactive oxidant molecules (two major components of the inflammatory process in the respiratory airways) suggests that erdosteine not only has mucolytic effects but may exert important anti-inflammatory effects in the airways of COPD patients.

In healthy smokers, acute smoke exposure decreased plasma nitrate plus nitrite concentrations and increased thiobarbituric acid reactive substances (TBARSs), markers of oxidative stress and lipid peroxidation, respectively. Short-term erdosteine (one month), while not changing nitrate/nitrite levels, significantly decreased TBARS concentrations, suggesting that erdosteine administration might help prevent smoking-induced lipid peroxidation [131]. Erdosteine was given at 600 mg/day for 10 days to current smokers with mild COPD, and its effect on pro-inflammatory and oxidative stress markers in bronchial secretions and blood was gauged against a placebo [132]. While no significant changes were observed in the placebo group, blood ROS and IL-8 in bronchial secretions dropped significantly following erdosteine starting from day 4, while the 8-isoprostane drop was significant only after day 10, and the e-NO decrease was evident but not significant. The same group aimed to assess the effect of antioxidant interventions on the short-term airway response to salbutamol in non-reversible mild-to-moderate COPD patients [133]. Erdosteine and NAC caused significant drops in ROS blood levels after four and ten days vs. the placebo. However, in contrast to NAC, erdosteine lowered 8-isoprostane levels substantially for ten days and was the only treatment to significantly restore short-term reversibility in COPD patients previously unresponsive to β2-adrenergics. Erdosteine treatment was also effective in reducing eicosanoids in COPD patients. Both LTB4 and LTE4 dropped significantly during the 10-day treatment with erdosteine. Moreover, a significant decrease in blood ROS was confirmed in patients treated with erdosteine. FEV_1_ (forced expiratory volume in the first second) values slightly increased during erdosteine treatment, with a significant difference in favor of erdosteine after 10 days of treatment.

In a single-center, double-blind, placebo-controlled study of patients with mild-to-moderate COPD (GOLD stage II-III), identifying the effects of two doses of erdosteine (600 and 900 mg/die) on oxidative stress, a reduction in the plasma levels of ROS and 8-isoprostane, a biomarker of oxidative stress, was found [134]. A dose-dependent decrease in ROS and 8-isoprostane plasma levels in both erdosteine-treated arms (600 and 900 mg/day) was studied. By week four, the 900 mg/day treatment proved to be more efficient than the 600 mg/day treatment. Interestingly, the effect of erdosteine was sustained and lasted over the post-treatment week. Moreover, the erdosteine treatment was shown to promote a systematic increase in FEV_1_ reversibility over the treatment period. The effect of erdosteine on exercise-induced oxidative stress was assessed by measuring and comparing the release of ROS and 8-isoprostane before and after exercise in patients with severe COPD receiving erdosteine (600 mg/day for 10 days) or a placebo [135]. At the end of the treatment period, after a 6 min walking test (6MWT), a significant difference in mean plasma ROS increase from baseline was noted between the erdosteine (+14.6% ± 2.7) and placebo groups (+24.4% ± 3.8) (*p* < 0.025). A similar significant trend was found for the mean 8-isoprostane increase.

A measure of systemic inflammation (C-reactive protein, CRP) was evaluated, alongside spirometric and exacerbation parameters, in exacerbated COPD patients who received erdosteine (900 mg/die), as an adjunctive treatment, or a placebo [136]. On day 10 of treatment, patients receiving erdosteine had lower levels of serum CRP. At the same time, clinical symptoms (breathlessness–sputum–cough scale) and lung function (FEV_1_) improved significantly compared to the placebo group. Erdosteine was associated with a 39.1% reduction in risk of exacerbation and a significant delay in time to first exacerbation.

### 5.4. Critical Appraisal and Translational Positioning of Erdosteine

Compared with several other oral mucolytics, erdosteine is supported by relatively consistent clinical data in COPD, yet the strength of the signal remains endpoint-dependent. The reduction in total and mild exacerbations appears more convincing than any effect on moderate-to-severe exacerbations, broad lung function improvement, or universally reproducible quality-of-life gains. This suggests a clinically relevant but circumscribed role, possibly in patients with recurrent mucus-rich exacerbations rather than across the full COPD spectrum. In addition, because erdosteine is a prodrug requiring hepatic metabolic activation, inter-individual variability in pharmacokinetic processing may contribute to differential responses. Taken together, the available evidence supports erdosteine as a plausible adjunctive option in selected patients but does not justify an undifferentiated indication across all COPD phenotypes.

## 6. Carbocisteine

### 6.1. Mechanisms of Action of Carbocisteine

Carbocisteine (CCis), i.e., S-Carboxymethylcysteine (SCMC) or its lysine salt, is a mucoactive agent, analog of NAC (Figure 2c), widely used to improve respiratory disease due to its multiple mechanisms of action that can help respiratory function and reduce inflammation. However, CCis does not bear free sulfhydryl (thiol) groups [137], and its clinical effects might be referred to a multipronged mechanism of action, including regulation of chloride and GSH secretion, balance of mucin expression, and antioxidant and anti-inflammatory properties [138]. Notably, the effects of CCis include the regulation of mucin production. In a mouse model of COPD, high doses of CCis have been shown to significantly decrease the expression of MUC5B and MUC5AC, restoring the Mus5b/Muc5ac ratio. This ratio was observed to be negatively correlated with pro-inflammatory cytokines (e.g., IL-6) but positively correlated with dynamic compliance [139]. CCis increased sialomucins while reducing fucomucins, likely via the intracellular stimulation of sialyl transferase activity, thus reducing mucus viscosity [140]. Further, it has been demonstrated that S-CMC-Lys normalized the viscous property of mucus by modifying fucosylated and sialylated sugar chains on the airway mucin MUC5AC [141]. Fucose and sialic acid contents on sugar chains on mucins are responsible for the viscous properties of mucus [142]. Thus, overall, the effects on chloride transport [143] and mucin production, structure, and balance may contribute to CCis’s mucoregulatory action and decrease mucus viscosity. In addition, one of its primary effects includes modulation of ciliary activity. CCis has been demonstrated to enhance the ciliary bend angle (CBA) and ciliary beat frequency (CBF) in mouse airway epithelial cells by decreasing the intracellular chloride concentration and increasing intracellular pH [144]. Similarly, in human nasal epithelial cells derived from subjects affected by chronic sinusitis and allergic rhinitis, CCis enhanced the ciliary beat amplitude, thereby promoting effective mucociliary clearance [145]. CCis has been shown to have antioxidant activity by improving GSH fluxes through cells. Garavaglia and colleagues [146] observed that the exposure of respiratory cells to the hydroxyl radical (·OH) caused irreversible inhibition of the GSH outward and chloride inward currents that was prevented if the cells were incubated with S-CMC-Lys. In cells lacking CFTR, this effect was not noted, suggesting that the restoration of GSH secretion by S-CMC-Lys is mediated by the CFTR channel [146,147]. However, it has been shown that the SCMC-Lys thioether group (R–S–R’) exerts selective scavenging activity towards hypochlorous acid (HOCl) and the hydroxyl radical (·OH), closely linked to cytoprotection and anti-inflammatory activity. Indeed, it reverses the inactivation of α1-antitrypsin and reduces the increased production of IL-8 due to the higher intracellular hydroxyl free radical activity [148]. Its cytoprotective actions are mediated by the phosphorylation of Akt, a well-known activator of prosurvival pathways [149]. A further significant effect of CCis is the modulation of the inflammatory response by reducing TNF-α-induced inflammation in alveolar epithelial cells. This is achieved by inhibiting the NF-κB and ERK1/2 MAPK signaling pathways, thereby reducing pro-inflammatory cytokine production, such as IL-6 and IL-8 [150].

### 6.2. Administration Route and Clinical Effects of Carbocisteine

Carbocisteine is typically administered orally in capsule form. The usual dosage is two 375 mg capsules three times a day, which has been demonstrated to be both effective and well tolerated in the management of COPD, with a minimal incidence of mild adverse reactions and with a substantial enhancement in quality of life [151].

To date, there are only a small number of clinical trials available that have tested the effects of CCis in patients with COPD [115,152,153,154]. The overall indication of these studies is that, due to variable and conflicting results, the anti-inflammatory and antioxidant properties of carbocisteine in COPD patients require time to be effective (at least one year). Thus, the longer the carbocisteine administration, the better the preventive effects against recurrent exacerbation [138]. In particular, a randomized controlled trial published in *The Lancet* in 2008 demonstrated that the administration of carbocisteine at a dosage of 1500 mg/day over a period of one year resulted in a reduction in the number of exacerbations [115]. Most recently, the clinical efficacy of CCis was investigated in a meta-analysis published in 2017 by Zeng et al. [155]. Analyzing 1357 patients obtained from four randomized controlled trials that met the inclusion criteria, the authors found that the long-term use of carbocisteine (500 mg TID) may be associated with lower exacerbation rates.

Patients with bronchiectasis were the focus of an observational, non-randomized, open study (a real-life study) with CCis vs. controls (those not receiving carbocisteine) over 3 months. The treated group had significantly fewer exacerbations than the control group, and, interestingly, the duration of exacerbations needed for complete resolution of symptoms or return of the symptoms to their baseline severity in the CCis-treated groups was significantly shorter than the mean duration of exacerbations in the placebo group [156]. However, further studies are needed to achieve more effective management of bronchiectasis, whether associated with COPD or not.

One early clinical study in CF demonstrated that SCMC-Lys was at least as effective as ambroxol in improving respiratory function in patients with cystic fibrosis. Notably, expectorate viscosity and elasticity decreased significantly in both groups [157]. The paucity of the literature on the effectiveness of thiol derivatives in CF warrants well-designed randomized placebo-controlled double-blind trials of people with this condition [158].

Carbocisteine is generally well tolerated, but it can cause some adverse effects, primarily related to the gastrointestinal system and the skin [159]. Patients may experience mild gastric discomfort such as stomach upset or irritation and, in rare cases, more severe complications like gastric ulceration, especially with prolonged use or higher doses. Additionally, fixed drug eruptions, a type of localized skin reaction that recurs at the same site upon re-exposure to the drug, have also been observed. However, three studies involving 1201 patients found no significant difference in the incidence of overall adverse events or gastrointestinal problems between CCis and placebo groups (RR 1.02, 95% CI 0.73–1.43, *p* = 0.75, and RR 1.38, 95% CI 0.71–2.66, *p* = 0.34, respectively) [153]. Importantly, no fatal adverse effects were reported, underscoring the relative safety of CCis when used appropriately, though monitoring for gastrointestinal and skin reactions remains advisable [155].

### 6.3. Immunomodulatory and Antioxidant Effects of Carbocisteine in Patients

In comparison with NAC, few studies have investigated the in vivo anti-inflammatory or antioxidant activities of carbocisteine in COPD patients. One study reported that exacerbated COPD patients treated with SCMC-Lys (2.7 g daily administrated orally) for 6 months showed a marked reduction in 8-isoprostane (an oxidative stress marker) and IL-6 in exhaled breath condensate [160]. More recently, a study presented the results obtained in mild AECOPD patients treated with CCis on various immune/inflammatory parameters: miR-21, IL-8, soluble Receptor for Advanced Glycation End Products (sRAGE), and fluorescent Advanced Glycation End Products (fAGEs). CCis not only improved symptoms and the pulmonary functional test but also increased circulating sRAGE, and reduced miR-21, IL-8, and fAGEs, indicating that CCIs may help to manage mild AECOPD by downregulating some parameters of systemic inflammation [161].

### 6.4. Critical Appraisal and Translational Positioning of Carbocisteine

Carbocisteine illustrates well the gap between mechanistic plausibility and clinical operationalization. Its proposed effects on mucin glycosylation, chloride transport, and ciliary function are biologically attractive, but their translation into consistently measurable clinical benefits remains supported by a relatively limited and heterogeneous evidence base. The available studies suggest that benefits are more likely to emerge with prolonged administration, which raises the possibility that carbocisteine acts less as an acute mucus “breaker” and more as a long-term mucoregulatory therapy. This temporal profile, together with its mechanistic features, supports the hypothesis that carbocisteine may be better suited to chronic bronchitic phenotypes characterized by persistent sputum production and altered mucus composition than to emphysema-predominant disease. However, the lack of routine biomarkers of mucin glycosylation or mucus subtypes remains a major obstacle to more precise clinical positioning.

## 7. Bromhexine

### 7.1. Mechanisms of Action of Bromhexine

Bromhexine (Figure 2d) is a mucoactive agent of synthetic origin, derived from vasicinone, a quinazoline alkaloid that exhibits bronchopulmonary dilation properties and is present in a variety of plant species, including *A. vasica* and *P. harmala* [162]. According to established pharmacological classifications, bromhexine is not a true mucolytic in the strict sense, as it does not directly cleave mucus polymers but rather acts as an expectorant and secretion-mobilizing agent that enhances mucociliary clearance. Bromhexine generally exists as the hydrochloride salt N-(2-amino-3,5-dibromobenzyl)-N-methyl-cyclo-hexanamine. Although the mechanisms of action of bromhexine are complex and have not yet been fully explored, it seems that its pharmacological activity is closely linked to the presence of two bromine atoms in positions 3 and 5 of the benzene ring. Together with the amino group, these atoms could be responsible for its secreto-motoric effect and modify the physicochemical characteristics of mucus to enhance mucociliary clearance [162,163]. Bromhexine acts primarily by depolymerizing mucopolysaccharide fibers within the mucus, thereby reducing its viscosity and facilitating its clearance from the respiratory tract. It enhances the production of less viscous secretions and stimulates the activity of lysosomal enzymes, which further degrade mucus components. Additionally, bromhexine increases the volume of bronchial secretions and improves mucociliary transport, contributing to its overall secretion-mobilizing and expectorant effects [162]. Recent research has identified bromhexine as a potent inhibitor of TMPRSS2, a transmembrane serine protease, which plays a significant role in the proteolytic cleavage of the SARS-CoV-2 “S” protein, facilitating the priming of ACE2 receptors and viral entry into human cells. This inhibitory activity is attributed to the compound’s ability to interact with the active site of the enzyme, potentially via the dibrominated benzyl group, which provides the necessary steric and electronic properties for binding [164]. Molecular docking studies suggest that binding affinity to viral targets could be influenced by specific structural elements, such as bromine atoms and amino groups [164].

A few preclinical studies have shown that bromhexine affects mucus production by altering the metabolism of alveolar type II cells, leading to increased secretion of phospholipids into the alveolar space [165]. In addition, bromhexine promotes mucociliary clearance, as demonstrated in an Italian multicenter study in which 237 patients with COPD were enrolled and treated either with a placebo or bromhexine [166]. Moreover, bromhexine significantly reduced the amount of mucopolysaccharide fibers, thereby reducing sputum viscosity [167].

Bromhexine was generally well tolerated across a wide range of clinical studies involving both adults and children with respiratory conditions. Most studies reported no significant adverse events. When adverse effects were noted, they were mild and infrequent, with the most common being gastrointestinal symptoms, headache, dizziness, and skin allergic reactions [162]. In the study by Nesswetha et al. [168], involving 242 patients with various respiratory conditions, bromhexine 5 mg administered three times daily for at least four days was well tolerated. No significant adverse effects were reported in any of the three double-blind, randomized controlled trials. Importantly, several large randomized controlled trials reported no adverse events at all, even at higher dosages and in vulnerable populations such as children [162].

### 7.2. Administration Route and Clinical Effects of Bromhexine

Bromhexine is widely available in the form of oral syrups and capsules. Some clinical trials have shown that oral bromhexine significantly increases sputum volume and decreases sputum viscosity, enhancing expectoration [167,169,170]. However, these initial studies have not yielded any significant improvements in overall respiratory function. Most studies conducted over the past years have been undertaken in patients diagnosed with chronic obstructive bronchitis. In an early double-blind, randomized, controlled trial, patients with mixed respiratory conditions (including chronic bronchitis) were treated with either bromhexine or a placebo [168]. The clinical endpoints that the authors evaluated included cough, sputum viscosity and disability. The results demonstrated only a reduction in the cough rate in the bromhexine treatment arm compared with the placebo group. Gent et al. conducted a cross-sectional, double-blind, placebo-controlled study to investigate the effects of bromhexine on pulmonary function in subjects who had been diagnosed with various lung diseases, including chronic bronchitis, asthma, emphysema and diffuse parenchymal lung disease, presenting with difficulty in expectorating sputum. The arm treated with bromhexine demonstrated clinical improvement in comparison to those treated with the placebo. However, functional residual capacity (FRC) and FEV_1_ measures were not significantly different between the two groups [171]. Subsequently, two studies demonstrated the efficacy of bromhexine in improving clinical outcomes in patients with chronic bronchitis. Specifically, a double-blind, randomized study evaluated the rheological mucus and pulmonary functional effects of bromhexine in 22 patients recovering from an exacerbation of chronic obstructive bronchitis. Compared to the placebo, data showed that bromhexine increased sputum production and reduced sputum viscosity, with no improvement in the overall respiratory state [172]. Christensen et al. [173] conducted a randomized, controlled trial on 61 patients diagnosed with chronic obstructive bronchitis and receiving either bromhexine or a placebo on a daily basis for a period of six months. The clinical endpoints indicated that the majority of patients experienced an improvement in symptoms and that the incidence of illness was lower among those who received bromhexine compared to those who received the placebo. An Italian multicenter, double-blind, randomized, controlled trial was conducted to evaluate the efficacy of bromhexine in patients with chronic obstructive lung disease [166]. The analysis of patient responses revealed a statistically significant decrease in cough and sputum volume, an improvement in expectoration, and decreased dyspnea in those treated with bromhexine compared to the placebo group. Furthermore, patients treated with bromhexine demonstrated a marked enhancement in pulmonary functional tests, in contrast to those who received the placebo.

In a recent meta-analysis of various mucolytics for acute exacerbations of COPD, Papadopoulou and colleagues [174] found no evidence that bromhexine ameliorates breathlessness, expectoration, or sputum viscosity. However, this evidence was obtained from the consideration of only two studies [175,176]. Only one study [177] compared oral bromhexine versus a placebo in the treatment of acute exacerbations of bronchiectasis with morning cough and purulent sputum. Participants in both treatment arms received an antibiotic. Difficulty in expectoration was significantly improved in the bromhexine-treated participants, and, notably, the quantity of sputum showed clinical improvement in bromhexine patients.

### 7.3. Antioxidant Effects of Bromhexine

Using pulse radiolysis, it was shown that ambroxol and bromhexine act as scavengers of both the superoxide anion and hydroxyl radicals, while NAC reacted with hydroxyl radicals but not with the superoxide anion [178].

### 7.4. Critical Appraisal and Translational Positioning of Bromhexine

Bromhexine is supported by a relatively modest and historically dated evidence base, with many studies relying on mixed respiratory populations, short-term outcomes, and symptom-oriented endpoints rather than contemporary exacerbation or quality-of-life measures. Its clinical effects on sputum properties and ease of expectoration appear to be more consistent than those on robust functional or long-term outcomes. Accordingly, bromhexine may retain a limited but plausible role as a secretion-mobilizing adjunct in chronic bronchitic states or selected exacerbations with retained sputum, whereas its positioning as a broadly effective mucolytic across muco-obstructive diseases is not supported by strong modern evidence.

## 8. Ambroxol

### 8.1. Mechanisms of Action of Ambroxol

Since the mid-twentieth century, the pharmaceutical agent ambroxol (2-amino-3,5-dibromo-N-[trans-4-hydroxy-cyclo-hexyl] benzylamine), a mucoactive agent and a metabolite of bromhexine (Figure 2e), has been used extensively in the treatment of diseases involving mucus obstruction. Ambroxol is classified as a mucoactive agent with expectorant and mucoregulatory properties rather than a classical mucus-cleaving mucolytic, as its primary actions involve stimulation of surfactant production, enhancement of mucociliary transport, and modulation of airway secretion. Ambroxol is distinguished from bromhexine by the absence of a methyl group and the introduction of a hydroxyl group in the para-trans position of the cyclohexyl ring [179]. The chemical structure of ambroxol, which features a dibrominated benzene ring with amino and hydroxy-cyclo-hexyl groups, enables multiple mechanisms of action, including sodium channel blockade and anti-inflammatory properties. A recent study has revealed that ambroxol activates voltage-dependent calcium channels in ciliated lung airway epithelial cells. This results in an increase in intracellular pH and a decrease in chloride concentrations, which ultimately enhances ciliary beat frequency and amplitude, thereby improving mucociliary clearance [180].

This molecule has been used for decades in managing both acute (such as bronchitis) and chronic (such as COPD) respiratory conditions [179,181,182]. This extensive use is due to its ability to stimulate mucociliary clearance and the production of endogenous surfactant, thus reducing mucus viscosity [183,184]. As compared to its parent compound bromhexine, the presence of a hydroxyl group in a para-trans position of the cyclohexyl ring and the deletion of a methyl group have provided ambroxol with additional pharmacological properties which make it able to stimulate surfactant production, reduce cytokine levels, and mitigate oxidative stress in the lungs [185]. Pharmacological and clinical research has demonstrated that ambroxol has mucoregulative and secretagogue effects [182,184]. Ambroxol promotes the synthesis and release of surfactant by type II pneumocytes and reduces sodium absorption in airway epithelial cells [186,187]. Furthermore, ambroxol has been demonstrated to exhibit direct scavenging properties towards reactive oxygen species and antioxidant properties, in addition to exerting anti-inflammatory effects through the inhibition of leukocytes from secreting pro-inflammatory mediators [184]. Anti-inflammatory and antioxidant properties of ambroxol have been studied in vitro and in vivo, using mouse models. One of the first articles regarding the role of ambroxol in the inhibition of inflammatory mechanisms was published in 1988 by Stockley et al. [188]. Human neutrophils were exposed to ambroxol at varying concentrations, and their migration toward chemotactic stimuli was assessed. The results showed that ambroxol significantly inhibited neutrophil chemotaxis in a dose-dependent manner, with up to 80% inhibition observed at 10^−4^ M, without inducing cytotoxicity. The authors concluded that ambroxol might exert an anti-inflammatory effect by limiting neutrophil recruitment to sites of inflammation, suggesting potential therapeutic relevance in conditions like chronic bronchitis and COPD. A study published by Zhang et al. [189] evaluated the effects of ambroxol administered via inhalation on LPS-induced airway inflammation and mucus hypersecretion in mice. The study also sought to understand the underlying mechanisms, focusing on the extracellular signal-regulated kinase 1/2 (ERK1/2) signaling pathway. Inhaled ambroxol (7.5 mg/mL) reduced MUC5AC expression and glycosaminoglycan levels in the lung tissues, enhancing mucociliary clearance and promoting sputum expectoration. Interestingly, in terms of anti-inflammatory actions, this study demonstrated that ambroxol diminished the influx of inflammatory cells and the expression of ERK1/2 in lung tissues. It also suppressed the mRNA expression of pro-inflammatory cytokines, including TNF-α, CCL-2, KC, and IL-1β. The study also compared the effects of inhaled and intravenous administration of the drug, providing insights into their relative efficacy in reducing airway inflammation and improving therapeutic outcomes. Notably, the effects of inhaled ambroxol were comparable to those of intravenous ambroxol (20 mg/mL) and intraperitoneal dexamethasone (0.5 mg/kg), indicating its potential as an effective inhalation therapy.

In order to elucidate the mechanism of action of ambroxol, its effects were evaluated in a human bronchial epithelial cell line (NCI-H292), showing that it inhibited LPS-induced increases in MUC5AC, TNF-α, and IL-1β mRNA expression by modulating the ERK signaling pathway. This study has evidenced that inhaled ambroxol effectively mitigates LPS-induced airway inflammation and mucus hypersecretion, primarily through the ERK1/2 signaling pathway. These findings support the potential of ambroxol as a therapeutic agent for airway inflammatory diseases. The anti-inflammatory effects of ambroxol are related to its ability to modulate multiple signaling pathways, including the NF-κB and Nrf2 pathways, through the dibrominated aromatic system that potentially contributes to antioxidant activity due to its electron-donating properties [190].

The antioxidant properties of ambroxol have been well recognized for many years. In vitro, ambroxol was shown to reduce ROS production in zymosan-activated human mononuclear and polymorphonuclear leukocytes. Its time-dependent increase in ROS reduction hints at additional cellular anti-inflammatory effects, possibly by modulating pro-oxidative metabolic pathways [191]. Ottonello et al. [192] investigated the capacity of ambroxol to inhibit neutrophil-mediated histotoxicity in vitro, proposing a multistep mechanism of action. The study demonstrated that ambroxol significantly reduced the production of superoxide anions and hypochlorous acid by activated human neutrophils. It also directly scavenged reactive oxygen species, limited the release of neutrophil-derived proteolytic enzymes such as elastase and MPO, and prevented the oxidative inactivation of alpha-1 antitrypsin. In contrast, its precursor, bromhexine, exhibited little to no activity in these assays. These findings suggest that ambroxol exerts a protective effect against neutrophil-driven oxidative and proteolytic tissue injury, highlighting its potential role as an adjunctive anti-inflammatory and antioxidant therapy in chronic inflammatory airway diseases. Additionally, ambroxol has been shown to exert antioxidant effects by scavenging reactive nitrogen species (RNS) and reducing nitrosative stress in the respiratory tract, thereby protecting airway epithelial cells and contributing to its therapeutic efficacy in inflammatory lung conditions [193]. In this context, a study published by Ricciardolo et al. [194] aimed to evaluate the effects of ambroxol and beclomethasone dipropionate (BDP) on LPS-induced nitrosative stress in human bronchial epithelial (BEAS-2B) cells, both in isolation and co-cultured with human polymorphonuclear neutrophils (PMNs). The study assessed the expression and release of various inflammatory markers, including RANTES, IL-8, inducible nitric oxide synthase (iNOS), MPO, and 3-nitrotyrosine (3-NT), a biomarker of nitrosative stress. In BEAS-2B cells alone, LPS exposure led to increased expression of RANTES (regulated upon activation, normal T cell expressed and secreted [also known as CCL5]) and iNOS, as well as elevated IL-8 levels. Ambroxol treatment suppressed RANTES expression and IL-8 release and inhibited iNOS expression. BDP reduced iNOS expression and IL-8 release but had no effect on RANTES levels. In BEAS-2B cells co-cultured with PMNs, LPS stimulation enhanced IL-8, MPO, and 3-NT production. Both ambroxol and BDP inhibited the release of these inflammatory markers. The combination of ambroxol and BDP further potentiated the inhibition of IL-8 and 3-NT production. The study concluded that ambroxol and BDP effectively inhibit nitrosative stress and the release of neutrophilic inflammatory products in vitro. The additive effects observed with their combination suggest potential therapeutic benefits in treating neutrophil-related respiratory diseases, such as COPD and respiratory infections. These studies collectively support the anti-inflammatory potential of ambroxol through various mechanisms, including cytokine modulation, oxidative stress reduction, and protection against neutrophil-mediated tissue damage.

### 8.2. Administration Route and Clinical Effects of Ambroxol

Ambroxol treatment is typically administered via inhalation or oral routes, with both methods being well established and effective. Intravenous (IV) administration of ambroxol is frequently employed in hospitalized patients who are unable to take oral or inhaled formulations [195,196]. Ambroxol is generally well tolerated, with a low incidence of adverse effects [196,197]. In 2004, a prospective, randomized, double-blind, placebo-controlled, multicenter parallel-group clinical trial was conducted to evaluate the long-term effect of ambroxol in preventing acute exacerbation in COPD patients [183]. Data demonstrated that patients diagnosed with COPD who were administered Ambroxol orally for a period of twelve months did not exhibit a substantial reduction in the incidence of acute exacerbations when compared to patients who received a placebo. However, a subgroup analysis of patients with more severe disease at baseline revealed a significant difference in cumulative exacerbation-free persistence, favoring ambroxol over the placebo. This finding suggests that the benefit of mucolytic treatment may be observed in COPD patients with more severe symptoms and repeated exacerbations [183]. In 2021, a study was conducted to investigate the effectiveness of ambroxol as an adjuvant treatment, in elderly patients suffering from COPD. The study population comprised 142 elderly COPD patients, who were divided into two groups: a control group and a research group. The control group received the standard symptomatic treatment, while the research group received ambroxol in addition to the treatment administered to the control group. The authors found that both treatments significantly reduced the sign score and the clinical symptoms exhibited by the patients, which included coughing, expectoration and dyspnea. Interestingly, the scores observed in the research group were consistently lower than those observed in the control group. In addition, the overall clinical efficacy rate was found to be significantly higher in the cohort of patients who were also treated with ambroxol in comparison to those treated solely with the standard treatment [197]. In 2024, the results of a prospective observational study aimed at examining the effectiveness of oral ambroxol and NAC administration in 60 COPD patients were published [198]. The study revealed no statistically significant differences in the average duration of hospitalization between patients treated with oral ambroxol and those treated with NAC. Conversely, a statistically significant enhancement in the percentage mean score of the peak expiratory flow rate was observed in both groups from admission to discharge. Interestingly, both groups demonstrated a marked improvement in symptoms such as breathlessness, cough and sputum production. In a recent study, hospitalized adult patients with mucopurulent sputum and expectoration difficulty (including those with AECOPD or bronchiectasis) were recruited and received inhaled ambroxol hydrochloride or a placebo for 5 days [196]. Ambroxol was significantly more effective than the placebo in reducing the sputum property score and also significantly reduced the expectoration volume in 24 h compared with the placebo. A limitation of this study was its short duration; thus, long-term trials are needed.

The therapeutic efficacy of oral NAC and ambroxol as compared with the effect of a placebo was studied in 36 CF patients with mild-to-moderate pulmonary disease [199]. Although no clinical differences could be observed in the two treated groups in comparison with the placebo, a significant increase in trapped air and a decrease in the FEV_1_ were found in the placebo group when compared to the active groups, suggesting a therapeutic effect of ambroxol and NAC in CF.

Ambroxol has been consistently reported to be well tolerated [184]. Adverse effects such as gastrointestinal disturbances and cutaneous rashes occurred only seldom, and they rarely led to treatment discontinuation. Additionally, in studies where laboratory monitoring was conducted, no adverse changes were observed following ambroxol treatment, further supporting the absence of clinically significant side effects. These findings reinforce the overall tolerability of ambroxol in adults [200]. Moreover, the robustness of available clinical data supports not only its efficacy but also its safety in pediatric populations, including infants and children with respiratory diseases, emphasizing ambroxol’s well-established safety across age groups.

### 8.3. Immunomodulatory and Antioxidant Effects of Ambroxol in Patients

Despite it being well known that ambroxol can stimulate surfactant production [184], increase mucociliary clearance by increasing ciliary beat frequency [201], and exert antioxidant and anti-inflammatory effects [202], scant information is available from clinical studies confirming these properties. Ambroxol-based adjuvant therapy was found to be effective in decreasing serum concentrations of IL-6, IL-8, and TNF-α as compared to a placebo group, thus indicating the potential of ambroxol to enhance patients’ inflammatory response [197]. Moreover, this study showed that the CD3+ and CD4+ levels in the ambroxol-treated patients were markedly higher than those in the control group, indicating that ambroxol as an adjuvant therapy could improve patients’ immune function.

### 8.4. Critical Appraisal and Translational Positioning of Ambroxol

Ambroxol occupies an intermediate position between classical mucolytics and broader mucoactive agents because its pharmacological profile includes secretolytic, surfactant-stimulating, antioxidant, and anti-inflammatory properties. However, the clinical evidence remains heterogeneous, with some signals of benefit in symptom control, sputum clearance, and selected COPD subgroups but limited consistency for major long-term outcomes across unselected populations. This suggests that ambroxol may be more relevant in mucus-rich phenotypes with impaired expectoration than as a universally effective therapy, although its broader biological actions justify further evaluation in stratified clinical settings.

## 9. Dornase Alfa

### 9.1. Mechanisms of Action of Dornase Alfa

Dornase alfa (Figure 2f), a recombinant human deoxyribonuclease (rhDNase) sold under the brand name Pulmozyme^®^, is primarily used as a peptide (260 amino acids) mucolytic to treat pulmonary disease in CF. An increase in polymerized deoxyribonucleic acid (DNA), together with F-actin released by neutrophils into the CF lung, is the leading cause of the tenacious and viscous properties of CF sputum [203]. It has been demonstrated that, upon administration, dornase reduces mucus viscosity by breaking down DNA present within secretions, “liquefying” the mucus-like structure, thereby promoting their clearance. DNase I also depolymerizes F-actin to G-actin [204], which is another putative mechanism for reducing CF sputum viscoelasticity [205]. The two primary goals of dornase alfa treatment are, firstly, to reduce the surface stickiness and viscosity of mucus and, secondly, to achieve mucolysis [206]. Exerting a different modality of action, the combination of dornase and NAC exhibits an additive effect on the increase in the transportability and decreased viscoelasticity of CF sputum [207].

### 9.2. Administration Route and Clinical Effects of Dornase Alfa

The drug is administered by nebulization or via an endotracheal tube. Over the last twenty years, more than fifteen clinical trials evaluating the effectiveness of DNase treatment in patients with CF and severe lung disease have been published. In 1994, Fuchs et al. [208] conducted a double-blind, randomized, placebo-controlled trial comparing the effectiveness of once-daily versus twice-daily DNase administration in 968 adults and children with CF over a period of 24 weeks. Patients treated with DNase once or twice daily experienced fewer respiratory exacerbations than those in the placebo group: 22% and 19%, respectively, compared to 27%. Administering DNase once or twice daily decreased the risk of respiratory exacerbation by 28% and 37%, respectively, and resulted in a significant increase in the FEV_1_ compared to the placebo group. In 1996, McCoy et al. conducted a multicenter, double-blind, randomized study in which 320 CF patients were enrolled and randomly divided into two groups: one group received dornase alfa treatment for 12 months, while the other received a placebo for the same period. Compared to the control group, the DNase-treated group showed increases in the FEV_1_ (9.4% vs. 2.1%) and an increase in the FVC (forced vital capacity) (12.4% vs. 7.3%). There were no differences in the number of days of antibiotic treatment, days of hospitalization, or adverse events between the two groups [209]. Quan et al. [210] studied the long-term effects of DNase treatment on spirometric parameters and respiratory exacerbations. The clinical study enrolled 239 children who were treated with a DNase-based drug and 235 children who were given a placebo. The FEV_1_, forced expiratory flow (FEF) 25–75% and FVC increased significantly in the DNase-treated group compared with the placebo one. Furthermore, the treatment with the DNase-based drug reduced the risk of respiratory exacerbation. No differences were observed in the risk of adverse events between the two groups. In 2006, Frederiksen et al. [211] evaluated the effect of a once-daily aerosolized DNase treatment on pulmonary colonization in patients with CF over a period of 12 months. The data demonstrated that the control group showed a significantly higher number of positive cultures, particularly for *Staphylococcus aureus*, compared to the drug-treated group. Similarly, patients treated with DNase showed an increase in the FEV_1_ compared to the placebo group (37% vs. 0.9%, respectively). In general, no increased risk of adverse events, such as hemoptysis, dyspnea or pneumothorax, was observed in CF patients who received DNase-based therapy compared to the control group [206,212].

Another important parameter that is more sensitive than spirometry for evaluating lung function outcomes is the lung clearance index (LCI). The LCI reflects global ventilation inhomogeneity and is primarily affected by small airway dysfunction. Amin et al. evaluated a DNase-based treatment in 17 pediatric patients with an FVC and FEV_1_ greater than 80%, over a period of 4 weeks. The data suggested that dornase alfa significantly improved the LCI compared with patients treated with the placebo [213]. A recent single-center, randomized, controlled, parallel-group study evaluated the effects of withdrawing nebulized DNase for one month in 28 children with CF. As the withdrawal period reached its conclusion, the authors noted a marked increase in the LCI, which was concomitant with a decline in both the FEV_1_ and FEF 25–75 values. These findings indicated an increase in ventilation inhomogeneity within the cohort of school-age children diagnosed with mild CF [214]. In conclusion, the DNase-based drug reduces the number of pulmonary exacerbations and improves both the FEV_1_ and LCI in patients with CF. Its ease of administration and good tolerability make it highly suitable for prescription [206]. Moreover, dornase alfa therapy is recommended for CF patients across all levels of disease severity [215].

However, it is important to note that the efficacy of dornase alfa is not uniform across all bronchiectasis cases. Specifically, in non-fibrocystic bronchiectasis, its administration has been associated with an elevated risk of exacerbations and a substantial decline in the FEV_1_.

Dornase alfa is generally well tolerated. The most commonly reported adverse reactions associated with dornase alfa include laryngitis, voice alteration, and rashes, occurring more frequently than with a placebo [206]. Other less common side effects reported are pharyngitis, chest pain, and conjunctivitis. These adverse effects are generally transient and mild, not severe enough to necessitate discontinuation of therapy or dosage adjustments. To date, there have been no reports of anaphylactic reactions linked to dornase alfa. Additionally, approximately 2 to 4% of patients treated with the drug have developed serum antibodies, although the clinical significance of these antibodies remains unclear. Rare reactions (under 1%) include urticaria and headache.

### 9.3. Anti-Inflammatory Effects of Dornase Alfa

An early study reported no effects of 1-month rhDNase treatment in sputum cytology of CF patients, although the administration of rhDNase resulted in significant improvements in the FEV_1_ [216]. On the other hand, in the Bronchoalveolar Lavage in the Evaluation of Anti-Inflammatory Treatment (BEAT) study, the treatment of CF patients with early lung disease with dornase alfa over 18 months reduced the DNA load in BAL fluid [217]. In the same study, BAL was also used to investigate the long-term effect of rhDNase on inflammation [218]. A significant increase in neutrophils was observed over the 3-year study period in both untreated patients and control subjects, whereas neutrophils remained unchanged in patients treated with rhDNase. Elastase activities and IL-8 concentrations also increased in untreated patients and remained stable in patients on rhDNase. This study demonstrates that rhDNase is not only an effective mucolytic drug but also affects the progression of airway inflammation in CF. The mechanism of the anti-inflammatory effect of rhDNase is unclear. As rhDNase has been found to improve clearance of airway mucus, this effect may directly clear neutrophils and their degradation products from the lung.

Metalloproteases (MMPs) can contribute to lung damage in CF. In a subset of the BEAT study, it was found that, 18 months after the first bronchoscopy, patients not receiving DNase showed an increase in MMP-8 and MMP-9 in BAL, whereas both MMP-8 and MMP-9 were decreased in DNase-treated patients [219]. Overall, dornase alfa can exert anti-inflammatory effects and may diminish long-term structural lung damage in CF; however, the precise mechanisms are not well known and might derive indirectly from its mucolytic activity [220].

### 9.4. Critical Appraisal and Translational Positioning of Dornase Alfa

Dornase alfa provides the clearest proof that mucolytic efficacy is maximized when the dominant rheological determinant of mucus is correctly targeted. In cystic fibrosis, where extracellular DNA substantially contributes to secretion tenacity and impaired clearance, recombinant DNase has demonstrated robust and reproducible benefits in lung function and exacerbation-related outcomes. By contrast, the lack of benefit—and potential harm—in non-CF bronchiectasis argues strongly against indiscriminate extrapolation across muco-obstructive diseases [30]. This divergence is biologically informative rather than disappointing: it indicates that mucus burden alone is not sufficient to predict response and that extracellular DNA must represent a major functional component of mucus pathology for dornase alfa to be beneficial. In this sense, dornase alfa in cystic fibrosis can be viewed as the current benchmark of successful target-matched mucolytic therapy within precision airway medicine.

## 10. Translational Stratification: From Pharmacological Classes to Mucus-Informed Use

A major reason for the inconsistent clinical performance of mucoactive agents is the mismatch between drug mechanisms and mucus composition. Disease labels such as COPD, bronchiectasis, or cystic fibrosis do not adequately capture the biological heterogeneity of airway secretions, which may differ in mucin concentration, disulfide cross-linking, extracellular DNA burden, infection status, hydration, and mucociliary transport. As a result, the same agent may perform differently across apparently similar clinical populations. Thiol-based agents such as NAC and erdosteine are most plausibly effective when mucus hyperviscosity is driven by mucin cross-linking and oxidative stress, as may occur in chronic bronchitis-predominant COPD or in frequent mucus-rich exacerbators. Carbocisteine may be better viewed as a long-term mucoregulatory therapy, potentially more suitable for patients with chronic productive cough, altered mucus composition, and impaired ciliary function than for emphysema-predominant disease. Bromhexine and ambroxol are more plausibly positioned in phenotypes characterized by impaired mucus transport and difficult expectoration, where secretion mobilization and improved mucociliary clearance may be more relevant than direct targeting of mucin cross-linking or extracellular DNA. Between the two, ambroxol may have broader translational appeal because, beyond secretolysis, it also shows surfactant-stimulating, antioxidant, and anti-inflammatory properties, whereas bromhexine seems to retain a narrower adjunctive role as a secretion-mobilizing agent. By contrast, dornase alfa remains the clearest example of target-matched mucolytic therapy: it is highly effective in cystic fibrosis, where extracellular DNA is a major determinant of mucus tenacity, but not in non-CF bronchiectasis, where mucus composition and airway pathobiology are different. This divergence highlights a key principle: mucus burden alone does not predict response; efficacy is greatest when the dominant biophysical determinant of secretion pathology is specifically targeted. Overall, these considerations support a shift from broad disease-label prescribing to mechanism-informed prescribing. Future mucolytic strategies should identify responsive subgroups according to mucus composition, inflammatory and infectious context, mucociliary dysfunction, and exacerbation profile, in line with the broader paradigm of precision airway medicine.

## 11. Challenges and Innovations in Mucolytic Therapy

One of the main challenges in mucolytic therapy is that clinical development has historically relied on broad diagnostic labels rather than on biologically informed patient selection. This has likely diluted treatment effects in trials that enrolled patients with markedly different mucus properties under the same disease umbrella. Future innovation should therefore not only aim at developing more potent or better-delivered compounds but also at identifying the patients most likely to benefit from each mechanism of action. In this respect, biomarkers reflecting mucus concentration, extracellular DNA burden, mucin subtype distribution, airway infection, and inflammatory phenotype may become essential tools for both clinical trials and routine implementation. To guide the discussion on challenges and innovations, we present below a comparative summary (Table 2) that aligns, for each agent, the primary mechanism of action, typical adult doses reported in the literature, target populations/settings, main outcomes (exacerbations, lung function, symptoms), trial durations, and key safety notes. This quick overview highlights where effects are most consistent and where gaps remain.

The therapy with mucoactive agents remains an essential component in the management of muco-obstructive lung diseases such as COPD, CF and bronchiectasis. Although thiol-reducing agents like NAC have long served as the cornerstone of mucolytic treatment, their clinical utility is constrained by several factors, including biochemical complexity, suboptimal pharmacokinetics, and disease-specific variability. A recent review has emphasized that the limited efficacy of NAC and related compounds stems from non-specific reduction of disulfide bonds and the inherent heterogeneity of mucus composition across different respiratory conditions [8]. The dense and complex architecture of mucus—comprising mucin glycoproteins, extracellular DNA, and actin—presents a formidable barrier to effective degradation, particularly when therapy is not precisely targeted.

Furthermore, the pharmacokinetic limitations of current mucolytics, including poor airway surface retention and low bioavailability, hinder their sustained therapeutic action. These challenges are compounded by the variability in mucus properties across disease states, which undermines the consistency of treatment outcomes. The indiscriminate mechanism of thiol-based agents may also lead to unintended interference with essential protein structures, contributing to their relatively modest clinical benefits.

In response to these limitations, novel mucolytic strategies are being developed to improve specificity, potency, and durability [221]. Researchers are increasingly focusing on agents that act beyond simple thiol-based reduction. New compounds targeting mucin cross-linking and mucin–DNA interactions aim to modulate the viscoelastic properties of mucus more selectively. Preclinical studies indicate that enzymatic cleavage of DNA (as achieved by dornase alfa) improves mucus transportability and airway clearance more effectively than non-specific thiol-based reduction when DNA burden is high [67,205]. Although no agents selectively disrupting mucin–DNA cross-linking are currently approved for clinical use, emerging preclinical approaches support the concept that targeting specific mucus components can yield superior clearance compared with broad mucolysis [87]. To the best of our knowledge, there are no specific, newly approved drugs exclusively targeting mucin polymerization that are in phase III trials for COPD. Despite this, a number of agents targeting mucus viscosity via the disruption of disulfide bonds are under investigation for COPD management. In diseases such as CF, recombinant human DNase (dornase alfa) has shown success by enzymatically degrading extracellular DNA, thereby reducing mucus viscosity. While its benefits in CF are well established, its utility in non-CF airway diseases is still being explored. Similarly, carbocisteine, a mucoregulator, exerts its effects by modifying the composition of mucin subtypes, particularly influencing the ratio of sialomucins to fucosomucins, which has implications for mucus rheology and clearance. Agents such as ambroxol and bromhexine offer additional mucokinetic benefits by stimulating surfactant production, enhancing mucociliary clearance, and promoting lysosomal activity in airway epithelial cells. These multifunctional properties position them as promising adjuncts in comprehensive airway management. Additionally, innovation in drug delivery systems is beginning to address pharmacokinetic shortcomings. Advanced inhalation formulations and nanoparticle-based carriers are being designed to enhance airway deposition, increase drug residence time, and improve mucolytic bioavailability at the site of action [222].

Erdosteine, a thiol-based prodrug with mucolytic, antioxidant, and anti-inflammatory properties, represents a further evolution of mucolytic therapy. It is metabolized in the liver to active metabolites that exert therapeutic effects; however, this requirement for metabolic activation introduces variability in clinical response. Differences in hepatic enzyme activity between individuals contribute to inconsistent drug efficacy, limiting its predictability and broad clinical utility. In COPD, while clinical studies have consistently shown that erdosteine can reduce the rate and duration of mild exacerbations, its efficacy in preventing or mitigating moderate-to-severe exacerbations remains less conclusive and warrants further investigation in large-scale, stratified trials [116,117]. Additional challenges include the limited understanding of the long-term safety and tolerability of erdosteine, particularly at higher dosages or when used in combination with other respiratory therapies. Larger, stratified clinical trials are needed to clarify its role in this context.

Additional concerns surrounding erdosteine include the lack of long-term safety data, particularly regarding its use at higher doses or in combination with other respiratory therapies. The development of patient-specific biomarkers may offer a pathway toward more personalized and effective use of erdosteine, enabling clinicians to predict treatment response and tailor dosing strategies accordingly. Moreover, direct comparisons with other thiol-based agents such as NAC and carbocisteine could provide important insights into the relative advantages of each therapy.

Regardless of the drugs to be used, an emerging strategy to improve drug delivery in pulmonary diseases such as COPD is represented by nanoparticle (NP) technology. NP use, despite being mainly in the preclinical research phases, holds great promise for improving the efficiency of drug delivery and minimizing side effects. Particularly attractive are inhaled delivery strategies due to the extensive surface area of the lungs (approximately 140 m^2^ at the alveolar level [223]), which ensures direct access to the sites of mucus production. Unfortunately, airway mucus constitutes a highly efficient barrier that critically limits the effectiveness of NP-based pulmonary drug delivery. As a viscoelastic hydrogel composed primarily of mucin glycoproteins, it is designed to trap and eliminate inhaled pathogens and particulates through a combination of steric obstruction and adhesive interactions. In line with its physiological role across mucosal tissues, mucus in the respiratory tract can both physically hinder the diffusion of nanoparticles—particularly for particles approaching or exceeding the mesh pore size—and chemically bind them via hydrophobic and electrostatic interactions, depending on their surface properties. Consequently, these barrier functions significantly reduce nanoparticle mobility, promote rapid mucociliary clearance, and ultimately limit local bioavailability and therapeutic efficacy in the lungs [224]. Thus, particularly attractive are approaches considering NPs equipped with mucus-penetrating moieties able to deliver drugs also in the inner part of the mucus layer. Moreover, NPs within the nanometer range have deep lung penetration capacity, thus enhancing drug absorption, bioavailability, solubility and diffusion kinetics [225]. Future clinical trials are needed to confirm the potential of NPs in the clinic.

Overall, these developments indicate that future progress in mucolytic therapy will depend not only on more effective compounds and delivery systems but also on biologically informed patient selection and stratified trial design.

## 12. Conclusions

The main lesson emerging from the available literature is not that mucoactive agents are broadly ineffective but that their efficacy is highly context-dependent and often obscured by biologically unselected trial populations. The clinical performance of these agents varies according to mucus composition, inflammatory and infectious context, disease phenotype, dose, and treatment duration. From this perspective, dornase alfa in cystic fibrosis represents the strongest example of a successful target-matched mucolytic therapy, whereas NAC, erdosteine, and carbocisteine appear more promising when interpreted as phenotype-dependent rather than universally active treatments. The field should therefore move from disease-label prescribing to mechanism-informed prescribing, in which mucus biology helps guide drug selection. Such a shift would not only improve the translational relevance of future trials but also provide a clearer conceptual framework for integrating mucolytics into precision airway medicine.

## Figures and Tables

**Table 1:** Physiological vs. pathological airway mucus.

**Feature**	**Physiological Mucus**	**Pathological Mucus**
Daily production	~2 L/day (mostly swallowed at night) [7,8]	Increased, visible accumulation in airways [7,8]
Composition	~97% water, 0.9% salt, ~2% macromolecules (mucins, antimicrobial proteins, peptides) [7,8]	Higher solids content (↑ mucins, DNA, actin, inflammatory proteins) [7,8]
Biophysical state	Hydrated, low-viscosity gel supporting mucociliary clearance [7,8]	Hyperconcentrated/dehydrated, ↑ viscosity and elasticity [6,7,8]
Mechanisms of regulation	Balance between Na^+^ absorption and Cl^−^ secretion maintains hydration [8]	Na^+^/Cl^−^ imbalance → dehydration; polymer osmotic compression of mucins [8]

**Table 2:** Comparative summary of key mucolytics.

**Agent**	**Mechanism (Primary)**	**Typical Dose (Adult)**	**Population/Setting**	**Main Outcomes**	**Duration in Trials**	**Safety Notes**
N-acetylcysteine (NAC)	Thiol mucolytic [75]; replenishes GSH [81]; antioxidant [76,77,78,79,80]	600 mg BID to 1200 mg/day (oral) [89]; nebulized varies [93]	COPD [89,90,91,92,93,114]; limited in CF [102,103,104,105]/bronchiectasis [101]	↓ exacerbations mainly at higher doses [83]; surrogate improvements [89]	3–12 months [89,93]	GI discomfort [89]; odor/taste when nebulized [98]
Erdosteine	Prodrug → active thiol (M1) [108]; mucolytic/antioxidant [109]/anti-adhesive [126,128]	300 mg BID (600 mg/day) oral [112,116,134]	COPD (moderate); limited elsewhere [116,118,134]	↓ total/mild exacerbations; ↓ duration; QoL benefit in some studies [111,113,116,117,118]	6–12 months [116,117]	Generally well tolerated [111,113,116,117]
Carbocisteine	Modulates mucin sialylation [140]; ↑ ciliary function [138,144,159]	750 mg TID (2250 mg/day) oral [150]	COPD [152,153,154,155]; observational in bronchiectasis [156]	↓ exacerbations with ≥12 months use [153,154,155]	6–18 months [154,155]	GI upset; rare rash [159]
Ambroxol/bromhexine	Mucokinetic; surfactant stimulation [162,179,183,184]	Ambroxol, 30 mg TID [182]; bromhexine, 8 mg TID [162]	Adjunctive symptomatic therapy [166,170,172,181,182,183,184]	↓ sputum viscosity/volume; limited hard outcomes [162,167,172,179,181,182,196]	Weeks–months [172,173,183,196]	Generally safe [162,172,196,197]
Dornase alfa	DNase I: reduces extracellular DNA viscosity [204,205]	2.5 mg nebulized once daily (CF) [210]	CF cornerstone [206,220]; not recommended in non-CF bronchiectasis	CF: ↑ FEV_1_/LCI [209,210,213], ↓ exacerbations [208]; BE non-CF: neutral/negative	Months–years [208,209,210,211]	Voice alteration, cough [206]

BE: bronchiectasis; BID: bis in die; CF: cystic fibrosis; COPD: chronic obstructive pulmonary disease; FEV_1_: forced expiratory volume in 1 s; GI: gastrointestinal tract; QoL: quality of life; TID: ter in die.

## Data Availability

No new data were created or analyzed in this study. Data sharing is not applicable to this article.

## Appendix A

### Mucus Characterization

Rheology (the study of flow and deformation of matter) provides a powerful framework to analyze the viscoelastic behavior of mucus. In lung diseases, changes in mucus concentration directly affect its rheological properties, as well as its ability to function as a lubricant and selective barrier [226]. By characterizing these changes, researchers can gain valuable insights into disease progression and assess the effectiveness of therapeutic interventions.

Traditional rheological measurements, often referred to as macro-rheology, are used to obtain quantitative information about the bulk properties of mucus. Typically, a sample of approximately 2 mL of mucus is placed between two parallel plates. Under the action of oscillatory (sinusoidal) stress, the upper plate starts oscillating, and the extent/speed of its oscillation, recorded by the rheometer, depends on the rheological properties (viscosity and elasticity) of the mucus. Among the many pieces of information that can be determined by this set-up, one of the most important is represented by the mechanical spectrum, i.e., the dependence of the elastic (or storage modulus, G′) and the viscous (or loss modulus, G″) moduli on the deformation frequency. Mechanical spectrum fitting by theoretical models [227], such as the generalized Maxwell model or Voigt models, allows for the determination of the relaxation spectrum and the shear modulus G. According to Flory theory [228] or to more sophisticated theories [229], G knowledge allows for the estimation of the average mesh size of the polymeric network [230]. This parameter is crucial for understanding how mucus structure and function are altered in pathological conditions like CF and COPD.

As mucus is a very complex fluid and little is known about its microscale architecture [231], not all the dynamics governed by supramolecular interactions could be experimentally accessible by macro-rheology. In this context, in the nineties, rheological properties were deduced by the response of the fluid to thermal fluctuations, as probed by the average motion of small particles dispersed within the fluid, to provide a close representation of the response of the bulk liquid to an imposed shear strain [232,233,234]. This technique, denominated micro-rheology, implies the determination of rheological properties at the microscopic level by following the movement of micro- or nanoparticles, so-called particle tracking. From these studies, technologies have continuously evolved, yet the fundamental physics underlying this approach remain unchanged: the bulk mechanical susceptibility of the fluid governs the response of a small particle subjected to thermal stochastic forces, resulting in Brownian motion. Techniques such as dynamic light scattering or fluorescence video microscopy analysis can be employed to measure the mean square displacement of a probe particle, ⟨Δr^2^(τ)⟩, and relate it to the viscoelastic moduli by applying the generalized Stokes–Einstein relation (Equation (A1)), which connects the viscoelastic spectrum G(s) to the Laplace transform of ⟨Δr^2^(τ)⟩:

(A1) $$G\left(s\right)\;=2k_{B}T/\left[3\pi \mathrm{as}<{\Delta r}^{2}>\right]$$

where T is the absolute temperature, kB is the Boltzmann constant (kBT is the thermal energy), a is the particle radius, and s is the complex Laplace frequency. By substituting s with iω, where i is an imaginary unit and ω is the angular frequency, the complex modulus can be expressed as follows [235]:

(A2) $$G^{*}\left(\omega \right)=G^{\prime }\left(\omega \right)\;+\;iG^{\prime \prime }\left(\omega \right)$$

The complex micro-viscosity, η*(ω), which incorporates both the viscous (G″) and elastic (G′) components, is given by the following: η*(ω) = ωG*(ω) [233]. The mesh size of the sputum hydrogel (ξ) can be estimated based on the measured G′ (Equation (A3)) [236] as follows:

(A3) $$\xi \;=\;{\left(k_{B}T/G^{\prime }\right)}^{1/3}$$

The displacement of inert particles through the mucus network provides insights into spatial heterogeneity, filtering effects, mesh size, and particle–mucus interactions. Numerous studies on lung diseases have shown that changes in microstructure may contribute to more frequent pulmonary exacerbations and accelerated decline in lung function [5,237]. Duncan et al. [231] found that a decrease in (Δr2(t)) is associated with a decrease in the FEV_1_, while ξ increases with rhDNase treatment.

Clearly, the choice of particle diameter is critical for the interpretation of micro-rheological data. If particles are larger than the characteristic length scale of heterogeneity within the gel and do not interact with its components, ⟨Δr^2^(t)⟩ can be directly related to linear viscoelastic measurements obtained via macro-rheology. In contrast, using particles of varying diameters allows for the comparison of multiple ⟨Δr^2^(t)⟩ values, providing information on the effective mesh size and the range of heterogeneity [14].

It is beneficial to integrate both macro- and micro-rheological approaches, as a gap often exists between penetration behavior at scales larger than a few micrometers and the structural characteristics of these complex fluids. Wagner et al. [14] hypothesized that the stiff regions of the mucin-dense phase dominate the mechanical properties observed in macro-rheology, while tracer particles are excluded from these regions and instead move within the softer portions of the heterogeneous mucin network, with a consequent underestimation of the viscoelastic properties. It is also worth considering that the micro-rheology approach relies on drawing conclusions from a short correlation time and a small observed area [238]. As a matter of fact, however, we were able to verify that one of the most important mucus nano-properties (i.e., mesh size distribution) determined by the macro-rheology approach is in perfect agreement with that obtained from the micro-rheology/particle tracking approach [239,240].

Another technique that can complement macro-rheology by analyzing larger sample volumes and providing insights into the micro–nanostructure of the network is Low-Field Nuclear Magnetic Resonance (LF-NMR). This method has proven particularly useful for characterizing mucus from sputum samples [240]. LF-NMR exploits the behavior of hydrogen nuclei (protons) in response to perturbations of an external static magnetic field, B_0_. In the presence of B_0_, hydrogen dipoles tend to align along its direction, resulting in a net magnetization vector M oriented along the *Z*-axis (i.e., the direction of B_0_).

When a radiofrequency pulse B_1_, applied in the XY plane perpendicular to B_0_, perturbs the system, M is rotated into the XY-plane. After the removal of B_1_, the system undergoes relaxation: the XY component of M (M_XY_) decays over time, while the Z component (M_Z_) gradually recovers. This relaxation process is influenced by the three-dimensional environment surrounding the hydrogen atoms, making it a valuable probe of micro-nanostructural features. In particular, interactions with solid surfaces, such as dispersed or solubilized polymeric chains, significantly reduce proton relaxation behavior.

The magnetization decay can be described by the solution of the magnetization diffusion equation proposed by Brownstein and Tarr [241]:

(A4) $$I\left(t\right)\;=\;\sum_{i=1}^{m}A_{i}e^{\left(-t/T_{2i}\right)}$$

where t is time, I(t) is the ratio between the time-dependent value of M_XY_ and its maximum value (M_XYmax_) occurring just after B_1_ removal, T_2i_ represents the ith spin–spin or transverse relaxation time, while A_i_ are “weights” proportional to the number of hydrogen atoms whose dipole relaxation is characterized by T_2i_. The average transverse relaxation time (T_2m_) depends on the ratio between the solid surface area (S), which correlates with the number of protons near solid interfaces (bound water), and the whole water volume (V), which reflects the total number of protons (bound plus unbound water). Thus, T_2m_ is inversely related to the solid content in sputum [242]:

(A5) $$\frac{1}{T_{2m}}=\frac{S}{V}M+\;\frac{1}{T_{2H2O}}$$

where T_2H2O_ is the relaxation time of hydrogen atoms far from any solid surface, while M is a parameter, called relaxivity, equal to the ratio between the thickness of the water layer near any solid surface (bound water) and the relaxation time of the hydrogen atoms therein contained [242].

In diseases such as CF and COPD, pathological increases in proteins, mucins, and other biopolymers are well documented [243]. Several studies have shown a correlation between T_2m_ values and the concentration of these components in CF sputum. Moreover, inverse correlations have been observed between T_2m_ and both systemic (e.g., CRP) and local inflammatory markers (e.g., IL-1β, TNF-α) [243,244]. Beyond Brownstein’s model, Scherer’s theory highlights how different spatial organizations of polymeric networks (e.g., cubic, tetrahedral, octahedral) affect the S/V ratio and, consequently, the T_2m_ values [245]. This relationship between network mesh size and T_2i_ has motivated the combined use of LF-NMR and rheology. Specifically, correlations were investigated between T_2_ and (1) sputum viscoelasticity, (2) the mucociliary clearability index (MCI)/cough clearability index (CCI), and (3) the sputum average mesh size, ξ_avg_ [239]. By fitting Equation (A4) to the experimental decay of M_XY_, a discrete or continuous relaxation spectrum can be obtained. When combined with rheological data, this allows for the conversion of the relaxation time spectrum into a mesh size distribution using the following equation [227]:

(A6) $$\xi_{i}\;=\;\xi_{\mathrm{avg}}\frac{\left({\left(\frac{1}{T_{2}}\right)}_{m}-\frac{1}{T_{2H_{2}O}}\right)}{\left(\frac{1}{T_{2i}}-\frac{1}{T_{2H_{2}O}}\right)}$$

where ξ_i_ is the mesh size characterized by the relaxation time T_2i_, and ξ_avg_ is the average mesh size determined by macro-rheological measurements. Notably, when comparing sputum from healthy individuals and CF patients, the mesh size range shifts from approximately 300–600 nm to 10–300 nm, respectively [227], which aligns well with values reported in the literature [67]. Thus, LF-NMR represents a valuable complementary technique to rheology for the microstructural characterization of mucus. Its ability to probe the local environment of hydrogen nuclei provides insights into the network architecture at the microscale. Moreover, LF-NMR measurements are straightforward and rapid: a 1 mL sample is simply transferred into an NMR tube, which is then inserted into a dedicated slot in the instrument, allowing the analysis to begin almost immediately. Both LF-NMR and conventional rheology analyze the entire sample volume, and when used in combination, they provide complementary insights into the micro- and macrostructural organization of mucus. While LF-NMR probes the molecular environment of hydrogen atoms, traditional rheology assesses the bulk mechanical properties of the sample. In contrast, micro-rheology, which is based on the mobility of embedded particles, offers a localized view of rheological properties and sample heterogeneity. Interestingly, Suk et al. [67]. estimated mucus mesh size through the penetration of microparticles of varying diameters, identifying a range of approximately 50–140 nm in sputum samples from CF patients, consistent with values derived from LF-NMR and rheological analyses [239,240].

As an application example, the following experimental observations illustrate how LF-NMR and rheology can detect structural changes induced by mucolytic agents such as NAC. We verified that NAC’s action on the polymeric network permeating the CF sputum can be detected by means of LF-NMR. Indeed, its addition as powder to two (N1, N2) sputum samples (NAC mass fraction concentration ω1 = 0.25 and ω2 = 0.21; T_2m−1_ = 1652 ms; T_2m−2_ = 1145 ms) resulted in a T_2m_ increase already after 30 min incubation at 37 °C (T_2m−1_ = 1916 ms; T_2m−2_ = 1619 ms). In the two samples, the T_2m_ increase was equal to 16% and 41%, respectively, compared to the original T_2m_ (untreated sample). Notably, NAC’s action (reduction in polymeric network connectivity, i.e., increase in the average mesh size) was more evident for sample N2 (41% T_2m_ increase), obtained from a COPD patient with worse clinical conditions (lower T_2m_). For both samples, T_2m_ underwent a small reduction (T_2m−1_ = 1856 ms; T_2m−2_ = 1512 ms) after 24 h following NAC addition (incubation at 37 °C), implying a slight tendency of the polymer network towards restructuring. To confirm the data obtained by LF-NMR, NAC (ω = 0.17) was added to another aliquot of sample N1. Our data showed that, after 30 min at 37 °C, there was a shift from gel-like rheological behavior (G′ > G″) to solution behavior (G′ < G″). This supports and confirms the concept that NAC induces connectivity impairment in the polymeric network.

## References

[1] Henke M.O., Ratjen F. (2007). Mucolytics in cystic fibrosis. Paediatr. Respir. Rev..

[2] King M., Rubin B.K. (2002). P harmacological approaches to discovery and development of new mucolytic agents. Adv. Drug Deliv. Rev..

[3] Roe T., Talbot T., Terrington I., Johal J., Kemp I., Saeed K., Webb E., Cusack R., Grocott M.P.W., Dushianthan A. (2025). Physiology and pathophysiology of mucus and mucolytic use in critically ill patients. Crit. Care.

[4] Bansil R., Turner B.S. (2018). The biology of mucus: Composition, synthesis and organization. Adv. Drug Deliv. Rev..

[5] Lai S.K., Wang Y.Y., Wirtz D., Hanes J. (2009). Micro- and macrorheology of mucus. Adv. Drug Deliv. Rev..

[6] Papi A., Avdeev S., Calverley P.M.A., Cordeiro C.R., Jesenak M., Koblizek V., Petkova D., Rogliani P., Tarraf H., Tzanakis N. (2020). Use of mucolytics in COPD: A Delphi consensus study. Respir. Med..

[7] Fahy J.V., Dickey B.F. (2010). Airway Mucus Function and Dysfunction. N. Engl. J. Med..

[8] Hill D.B., Button B., Rubinstein M., Boucher R.C. (2022). PHYSIOLOGY AND PATHOPHYSIOLOGY OF HUMAN AIRWAYMUCUS. Physiol. Rev..

[9] Shah B.K., Singh B., Wang Y., Xie S., Wang C. (2023). Mucus Hypersecretion in Chronic Obstructive Pulmonary Disease and Its Treatment. Mediat. Inflamm..

[10] Perez J., Hill R. (2004). Mucin Family of Glycoproteins. Encycl. Biol. Chem..

[11] Snary D., Allen A., Pain R.H. (1970). Structural studies on gastric mucoproteins: Lowering of molecular weight after reduction with 2-mercaptoethanol. Biochem. Biophys. Res. Commun..

[12] Gillis R.B., Adams G.G., Wolf B., Berry M., Besong T.M.D., Corfield A., Kök S.M., Sidebottom R., Lafond D., Rowe A.J. (2013). Molecular weight distribution analysis by ultracentrifugation: Adaptation of a new approach for mucins. Carbohydr. Polym..

[13] Carpenter J., Wang Y., Gupta R., Li Y., Haridass P., Subramani D.B., Reidel B., Morton L., Ridley C., O’Neal W.K. (2021). Assembly and organization of the N-terminal region of mucin MUC5AC: Indications for structural and functional distinction from MUC5B. Proc. Natl. Acad. Sci. USA.

[14] Wagner C.E., Turner B.S., Rubinstein M., McKinley G.H., Ribbeck K. (2017). A Rheological Study of the Association and Dynamics of MUC5AC Gels. Biomacromolecules.

[15] Cao X., Bansil R., Bhaskar K.R., Turner B.S., LaMont J.T., Niu N., Afdhal N.H. (1999). pH-dependent conformational change of gastric mucin leads to sol-gel transition. Biophys. J..

[16] Ostedgaard L.S., Moninger T.O., McMenimen J.D., Sawin N.M., Parker C.P., Thornell I.M., Powers L.S., Gansemer N.D., Bouzek D.C., Cook D.P. (2017). Gel-forming mucins form distinct morphologic structures in airways. Proc. Natl. Acad. Sci. USA.

[17] Erken O., Fazla B., Muradoglu M., Izbassarov D., Romanò F., Grotberg J.B. (2023). Effects of elastoviscoplastic properties of mucus on airway closure in healthy and pathological conditions. Phys. Rev. Fluids.

[18] Bansil R., Turner B.S. (2006). Mucin structure, aggregation, physiological functions and biomedical applications. Curr. Opin. Colloid Interface Sci..

[19] Franzino G., Tescione F., Larobina D. (2025). Macroheterogeneities Induced by Disulfide Bond Reduction in Native Mucus. ACS Appl. Bio Mater..

[20] Meldrum O.W., Yakubov G.E., Bonilla M.R., Deshmukh O., McGuckin M.A., Gidley M.J. (2018). Mucin gel assembly is controlled by a collective action of non-mucin proteins, disulfide bridges, Ca^2+^ -mediated links, and hydrogen bonding. Sci. Rep..

[21] Shih C.K., Litt M., Khan M.A., Wolf D.P. (1977). Effect of nondialyzable solids concentration and viscoelasticity on ciliary transport of tracheal mucus. Am. Rev. Respir. Dis..

[22] Hill D.B., Vasquez P.A., Mellnik J., McKinley S.A., Vose A., Mu F., Henderson A.G., Donaldson S.H., Alexis N.E., Boucher R.C. (2014). A biophysical basis for mucus solids concentration as a candidate biomarker for airways disease. PLoS ONE.

[23] Bush A., Payne D., Pike S., Jenkins G., Henke M.O., Rubin B.K. (2006). Mucus properties in children with primary ciliary dyskinesia: Comparison with cystic fibrosis. Chest.

[24] Rogers G.B., Carroll M.P., Zain N.M.M., Bruce K.D., Lock K., Walker W., Jones G., Daniels T.W.V., Lucas J.S. (2013). Complexity, temporal stability, and clinical correlates of airway bacterial community composition in primary ciliary Dyskinesia. J. Clin. Microbiol..

[25] Sears P.R., Bustamante-Marin X.M., Gong H., Markovetz M.R., Superfine R., Hill D.B., Ostrowski L.E. (2021). Induction of ciliary orientation by matrix patterning and characterization of mucociliary transport. Biophys. J..

[26] Rouillard K.R., Kissner W.J., Markovetz M.R., Hill D.B. (2022). Effects of Mucin and DNA Concentrations in Airway Mucus on Pseudomonas aeruginosa Biofilm Recalcitrance. mSphere.

[27] Yuan S., Hollinger M., Lachowicz-Scroggins M.E., Kerr S.C., Dunican E.M., Daniel B.M., Ghosh S., Erzurum S.C., Willard B., Hazen S.L. (2015). Oxidation increases mucin polymer cross-links to stiffen airway mucus gels. Sci. Transl. Med..

[28] Järvå M.A., Lingford J.P., John A., Soler N.M., Scott N.E., Goddard-Borger E.D. (2020). Trefoil factors share a lectin activity that defines their role in mucus. Nat. Commun..

[29] Bastholm S.K., Samson M.H., Becher N., Hansen L.K., Stubbe P.R., Chronakis I.S., Nexo E., Uldbjerg N. (2017). Trefoil factor peptide 3 is positively correlated with the viscoelastic properties of the cervical mucus plug. Acta Obstet. Gynecol. Scand..

[30] Chang A.B., Bush A., Grimwood K. (2018). Bronchiectasis in children: Diagnosis and treatment. Lancet.

[31] Mall M.A., Burgel P.R., Castellani C., Davies J.C., Salathe M., Taylor-Cousar J.L. (2024). Cystic fibrosis. Nat. Rev. Dis. Prim..

[32] Barnes P.J., Burney P.G., Silverman E.K., Celli B.R., Vestbo J., Wedzicha J.A., Wouters E.F. (2015). Chronic obstructive pulmonary disease. Nat. Rev. Dis. Prim..

[33] De Rose V., Molloy K., Gohy S., Pilette C., Greene C.M. (2018). Airway Epithelium Dysfunction in Cystic Fibrosis and COPD. Mediat. Inflamm..

[34] Mall M.A., Criner G.J., Miravitlles M., Rowe S.M., Vogelmeier C.F., Rowlands D.J., Schoenberger M., Altman P. (2023). Cystic fibrosis transmembrane conductance regulator in COPD: A role in respiratory epithelium and beyond. Eur. Respir. J..

[35] Raju S.V., Solomon G.M., Dransfield M.T., Rowe S.M. (2016). Acquired Cystic Fibrosis Transmembrane Conductance Regulator Dysfunction in Chronic Bronchitis and Other Diseases of Mucus Clearance. Clin. Chest Med..

[36] Mall M.A., Hartl D. (2014). CFTR: Cystic fibrosis and beyond. Eur. Respir. J..

[37] Mall M.A. (2008). Role of cilia, mucus, and airway surface liquid in mucociliary dysfunction: Lessons from mouse models. J. Aerosol Med. Pulm. Drug Deliv..

[38] Clunes L.A., Davies C.M., Coakley R.D., Aleksandrov A.A., Henderson A.G., Zeman K.L., Worthington E.N., Gentzsch M., Kreda S.M., Cholon D. (2012). Cigarette smoke exposure induces CFTR internalization and insolubility, leading to airway surface liquid dehydration. FASEB J..

[39] Raju S.V., Jackson P.L., Courville C.A., McNicholas C.M., Sloane P.A., Sabbatini G., Tidwell S., Tang L.P., Liu B., Fortenberry J.A. (2013). Cigarette smoke induces systemic defects in cystic fibrosis transmembrane conductance regulator function. Am. J. Respir. Crit. Care Med..

[40] Raju S.V., Lin V.Y., Liu L., McNicholas C.M., Karki S., Sloane P.A., Tang L., Jackson P.L., Wang W., Wilson L. (2017). The Cystic Fibrosis Transmembrane Conductance Regulator Potentiator Ivacaftor Augments Mucociliary Clearance Abrogating Cystic Fibrosis Transmembrane Conductance Regulator Inhibition by Cigarette Smoke. Am. J. Respir. Cell Mol. Biol..

[41] Mazumdar M., Christiani D.C., Biswas S.K., Ibne-Hasan O.S., Kapur K., Hug C. (2015). Elevated sweat chloride levels due to arsenic toxicity. N. Engl. J. Med..

[42] Hassan F., Nuovo G.J., Crawford M., Boyaka P.N., Kirkby S., Nana-Sinkam S.P., Cormet-Boyaka E. (2012). MiR-101 and miR-144 regulate the expression of the CFTR chloride channel in the lung. PLoS ONE.

[43] Viart V., Bergougnoux A., Bonini J., Varilh J., Chiron R., Tabary O., Molinari N., Claustres M., Taulan-Cadars M. (2015). Transcription factors and miRNAs that regulate fetal to adult CFTR expression change are new targets for cystic fibrosis. Eur. Respir. J..

[44] Le Gars M., Descamps D., Roussel D., Saussereau E., Guillot L., Ruffin M., Tabary O., Hong S.S., Boulanger P., Paulais M. (2013). Neutrophil elastase degrades cystic fibrosis transmembrane conductance regulator via calpains and disables channel function in vitro and in vivo. Am. J. Respir. Crit. Care Med..

[45] McKelvey M.C., Brown R., Ryan S., Mall M.A., Weldon S., Taggart C.C. (2021). Proteases, Mucus, and Mucosal Immunity in Chronic Lung Disease. Int. J. Mol. Sci..

[46] Downs C.A., Kreiner L.H., Trac D.Q., Helms M.N. (2013). Acute effects of cigarette smoke extract on alveolar epithelial sodium channel activity and lung fluid clearance. Am. J. Respir. Cell Mol. Biol..

[47] Barnes P.J. (2022). Oxidative Stress in Chronic Obstructive Pulmonary Disease. Antioxidants.

[48] Moliteo E., Sciacca M., Palmeri A., Papale M., Manti S., Parisi G.F., Leonardi S. (2022). Cystic Fibrosis and Oxidative Stress: The Role of CFTR. Molecules.

[49] Linssen R.S., Chai G., Ma J., Kummarapurugu A.B., van Woensel J.B.M., Bem R.A., Kaler L., Duncan G.A., Zhou L., Rubin B.K. (2021). Neutrophil Extracellular Traps Increase Airway Mucus Viscoelasticity and Slow Mucus Particle Transit. Am. J. Respir. Cell Mol. Biol..

[50] Conese M., Castellani S., D’Oria S., di Gioia S., Montemurro P., Khajah M.A. (2017). Role of Neutrophils in Cystic Fibrosis Lung Disease. Role of Neutrophils in Disease Pathogenesis.

[51] Button B., Cai L.H., Ehre C., Kesimer M., Hill D.B., Sheehan J.K., Boucher R.C., Rubinstein M. (2012). A periciliary brush promotes the lung health by separating the mucus layer from airway epithelia. Science.

[52] Mullen E., Murphy M., Bateman G., Casey M., Gunaratnam C., McElvaney N.G. (2025). An update on targeting airway inflammation in cystic fibrosis. Expert Rev. Respir. Med..

[53] Ghigo A., Prono G., Riccardi E., De Rose V. (2021). Dysfunctional Inflammation in Cystic Fibrosis Airways: From Mechanisms to Novel Therapeutic Approaches. Int. J. Mol. Sci..

[54] Terlizzi V., Masi E., Francalanci M., Taccetti G., Innocenti D. (2021). Hypertonic saline in people with cystic fibrosis: Review of comparative studies and clinical practice. Ital. J. Pediatr..

[55] Grigoletto A., Mori G., Delfino D., Bizzotto G., Campara B., Calienni M.N., Buttini F., Glieca S., Rosa G.D., Tedeschini T. (2026). Therapeutic potential of a long-acting form of rhDNase I for the treatment of cystic fibrosis. Int. J. Biol. Macromol..

[56] Zimmermann C.M., Baldassi D., Chan K., Adams N.B.P., Neumann A., Porras-Gonzalez D.L., Wei X., Kneidinger N., Stoleriu M.G., Burgstaller G. (2022). Spray drying siRNA-lipid nanoparticles for dry powder pulmonary delivery. J. Control. Release.

[57] Mall M.A., Danahay H., Boucher R.C. (2018). Emerging Concepts and Therapies for Mucoobstructive Lung Disease. Ann. Am. Thorac. Soc..

[58] Lee A.Y., Cho M.H., Kim S. (2019). Recent advances in aerosol gene delivery systems using non-viral vectors for lung cancer therapy. Expert Opin. Drug Deliv..

[59] Liu Y., Sukumar U.K., Jugniot N., Seetharam S.M., Rengaramachandran A., Sadeghipour N., Mukherjee P., Krishnan A., Massoud T.F., Paulmurugan R. (2022). Inhaled Gold Nano-star Carriers for Targeted Delivery of Triple Suicide Gene Therapy and Therapeutic MicroRNAs to Lung Metastases: Development and Validation in a Small Animal Model. Adv. Ther..

[60] Puccetti M., Pariano M., Stincardini C., Wojtylo P., Schoubben A., Nunzi E., Ricci M., Romani L., Giovagnoli S. (2023). Pulmonary drug delivery technology enables anakinra repurposing in cystic fibrosis. J. Control. Release.

[61] Kokkinis S., Singh M., Paudel K.R., De Rubis G., Bani Saeid A., Jessamine V., Datsyuk J., Singh S.K., Vishwas S., Adams J. (2024). Plant-based therapeutics for chronic obstructive pulmonary diseases: Nanoformulation strategies to overcome delivery challenges. Food Biosci..

[62] Sonntag T., Rapp M., Didier P., Lebeau L., Pons F., Casset A. (2022). Mucus-producing epithelial models for investigating the activity of gene delivery systems in the lung. Int. J. Pharm..

[63] Anjum M.M., Patel K.K., Bhattacharya S., Arya D.K., Pandey P., Mr V., Singh S., Rajinikanth P.S. (2023). Overcoming barriers in cystic fibrosis therapy through inhalational lipid nanoparticles: Challenges and advances. J. Drug Deliv. Sci. Technol..

[64] Taghavizadeh Yazdi M.E., Qayoomian M., Beigoli S., Boskabady M.H. (2023). Recent advances in nanoparticle applications in respiratory disorders: A review. Front. Pharmacol..

[65] Button B., Okada S.F., Frederick C.B., Thelin W.R., Boucher R.C. (2013). Mechanosensitive ATP release maintains proper mucus hydration of airways. Sci. Signal..

[66] Abrami M., Biasin A., Maschio M., Conese M., Confalonieri M., Gerin F., Salton F., Confalonieri P., Ruaro B., Casalaz R. (2025). Indirect evaluation of lung condition by means of LF-NMR following chest physiotherapy or ETI administration in cystic-fibrosis patients. Heart Lung.

[67] Suk J.S., Lai S.K., Wang Y.Y., Ensign L.M., Zeitlin P.L., Boyle M.P., Hanes J. (2009). The penetration of fresh undiluted sputum expectorated by cystic fibrosis patients by non-adhesive polymer nanoparticles. Biomaterials.

[68] Grassi M., Grassi G., Lapasin R., Colombo I. (2007). Understanding Drug Release and Absorption Mechanisms: A physical and Mathematical Approach.

[69] Rano S., Bhaduri A., Singh M. (2024). Nanoparticle-based platforms for targeted drug delivery to the pulmonary system as therapeutics to curb cystic fibrosis: A review. J. Microbiol. Methods.

[70] Ghanem R., Berchel M., Haute T., Buin X., Laurent V., Youf R., Bouraoui A., Le Gall T., Jaffres P.A., Montier T. (2023). Gene transfection using branched cationic amphiphilic compounds for an aerosol administration in cystic fibrosis context. Int. J. Pharm..

[71] Liu Z., Che B., Zhang H., Deng L. (2025). Airway Mucus Rheology: Physical Insights for Navigating through Health to Pathology and Clinical Applications. arXiv.

[72] Poole P., Sathananthan K., Fortescue R. (2019). Mucolytic agents versus placebo for chronic bronchitis or chronic obstructive pulmonary disease. Cochrane Database Syst. Rev..

[73] D’Antonio S., Pennisi A., Cazzola M. (2025). Mucolytic Therapy in COPD: Patient Usage and Preferences in Real-World Italian Settings. Int. J. Chronic Obstr. Pulm. Dis..

[74] Hurst G.A., Shaw P.B., LeMaistre C.A. (1967). Laboratory and clinical evaluation of the mucolytic properties of acetylcysteine. Am. Rev. Respir. Dis..

[75] Rogliani P., Manzetti G.M., Gholamalishahi S., Cazzola M., Calzetta L. (2024). Impact of N-Acetylcysteine on Mucus Hypersecretion in the Airways: A Systematic Review. Int. J. Chronic Obstr. Pulm. Dis..

[76] Aldini G., Altomare A., Baron G., Vistoli G., Carini M., Borsani L., Sergio F. (2018). N-Acetylcysteine as an antioxidant and disulphide breaking agent: The reasons why. Free Radic. Res..

[77] Schwalfenberg G.K. (2021). N-Acetylcysteine: A Review of Clinical Usefulness (an Old Drug with New Tricks). J. Nutr. Metab..

[78] Tenorio M., Graciliano N.G., Moura F.A., Oliveira A.C.M., Goulart M.O.F. (2021). N-Acetylcysteine (NAC): Impacts on Human Health. Antioxidants.

[79] Mokra D., Mokry J., Barosova R., Hanusrichterova J. (2023). Advances in the Use of N-Acetylcysteine in Chronic Respiratory Diseases. Antioxidants.

[80] Santus P., Signorello J.C., Danzo F., Lazzaroni G., Saad M., Radovanovic D. (2024). Anti-Inflammatory and Anti-Oxidant Properties of N-Acetylcysteine: A Fresh Perspective. J. Clin. Med..

[81] De Benedetto F., Aceto A., Dragani B., Spacone A., Formisano S., Pela R., Donner C.F., Sanguinetti C.M. (2005). Long-term oral n-acetylcysteine reduces exhaled hydrogen peroxide in stable COPD. Pulm. Pharmacol. Ther..

[82] Drost E.M., Skwarski K.M., Sauleda J., Soler N., Roca J., Agusti A., MacNee W. (2005). Oxidative stress and airway inflammation in severe exacerbations of COPD. Thorax.

[83] Papi A., Alfano F., Bigoni T., Mancini L., Mawass A., Baraldi F., Aljama C., Contoli M., Miravitlles M. (2024). N-acetylcysteine Treatment in Chronic Obstructive Pulmonary Disease (COPD) and Chronic Bronchitis/Pre-COPD: Distinct Meta-analyses. Arch. Bronconeumol..

[84] Cazzola M., Calzetta L., Facciolo F., Rogliani P., Matera M.G. (2017). Pharmacological investigation on the anti-oxidant and anti-inflammatory activity of N-acetylcysteine in an ex vivo model of COPD exacerbation. Respir. Res..

[85] Calzetta L., Rogliani P., Facciolo F., Rinaldi B., Cazzola M., Matera M.G. (2018). N-Acetylcysteine protects human bronchi by modulating the release of neurokinin A in an ex vivo model of COPD exacerbation. Biomed. Pharmacother..

[86] Montero P., Roger I., Estornut C., Milara J., Cortijo J. (2023). Influence of dose and exposition time in the effectiveness of N-Acetyl-l-cysteine treatment in A549 human epithelial cells. Heliyon.

[87] Ehre C., Rushton Z.L., Wang B., Hothem L.N., Morrison C.B., Fontana N.C., Markovetz M.R., Delion M.F., Kato T., Villalon D. (2019). An Improved Inhaled Mucolytic to Treat Airway Muco-obstructive Diseases. Am. J. Respir. Crit. Care Med..

[88] Sadowska A.M., Manuel Y.K.B., De Backer W.A. (2007). Antioxidant and anti-inflammatory efficacy of NAC in the treatment of COPD: Discordant in vitro and in vivo dose-effects: A review. Pulm. Pharmacol. Ther..

[89] Tse H.N., Raiteri L., Wong K.Y., Yee K.S., Ng L.Y., Wai K.Y., Loo C.K., Chan M.H. (2013). High-dose N-acetylcysteine in stable COPD: The 1-year, double-blind, randomized, placebo-controlled HIACE study. Chest.

[90] Zhou Y., Wu F., Shi Z., Cao J., Tian J., Yao W., Wei L., Li F., Cai S., Shen Y. (2024). Effect of high-dose N-acetylcysteine on exacerbations and lung function in patients with mild-to-moderate COPD: A double-blind, parallel group, multicentre randomised clinical trial. Nat. Commun..

[91] Tang W., Zhu D., Wu F., Xu J.F., Yang J.P., Deng Z.P., Chen X.B., Papi A., Qu J.M. (2023). Intravenous N-acetylcysteine in respiratory disease with abnormal mucus secretion. Eur. Rev. Med. Pharmacol. Sci..

[92] App E.M., Baran D., Dab I., Malfroot A., Coffiner M., Vanderbist F., King M. (2002). Dose-finding and 24-h monitoring for efficacy and safety of aerosolized Nacystelyn in cystic fibrosis. Eur. Respir. J..

[93] Rhee C.K., Lim S.Y., Lee W.Y., Jung J.Y., Park Y.B., Lee C.Y., Hwang Y.I., Song J.W., Choi W.I., Yoo K.H. (2024). The effect of nebulized N-acetylcysteine on the phlegm of chronic obstructive pulmonary disease: The NEWEST study. BMC Pulm. Med..

[94] Banerjee S., McCormack S. (2019). Acetylcysteine for Patients Requiring Mucous Secretion Clearance: A Review of Clinical Effectiveness and Safety.

[95] Bebarta V.S., Kao L., Froberg B., Clark R.F., Lavonas E., Qi M., Delgado J., McDonagh J., Arnold T., Odujebe O. (2010). A multicenter comparison of the safety of oral versus intravenous acetylcysteine for treatment of acetaminophen overdose. Clin. Toxicol..

[96] Holdiness M.R. (1991). Clinical pharmacokinetics of N-acetylcysteine. Clin. Pharmacokinet..

[97] Mahmoudi G.A., Astaraki P., Mohtashami A.Z., Ahadi M. (2015). N-acetylcysteine overdose after acetaminophen poisoning. Int. Med. Case Rep. J..

[98] Ohnishi H., Tanimoto T., Inaba R., Eitoku M. (2024). Efficacy and safety of mucolytics in patients with stable chronic obstructive pulmonary disease: A systematic review and meta-analysis. Respir. Investig..

[99] Hocquigny A., Hugerot H., Ghanem R., Haute T., Laurent V., Cogulet V., Montier T. (2024). Mucoactive drugs and multiple applications in pulmonary disease therapy. Eur. J. Pharm. Biopharm..

[100] De Backer J., Vos W., Van Holsbeke C., Vinchurkar S., Claes R., Parizel P.M., De Backer W. (2013). Effect of high-dose N-acetylcysteine on airway geometry, inflammation, and oxidative stress in COPD patients. Int. J. Chronic Obstr. Pulm. Dis..

[101] Jayaram L., King P.T., Hunt J., Lim M., Park C., Hu E., Dousha L., Ha P., Bartlett J.B., Southcott A.M. (2024). Evaluation of high dose N- Acetylcysteine on airway inflammation and quality of life outcomes in adults with bronchiectasis: A randomised placebo-controlled pilot study. Pulm. Pharmacol. Ther..

[102] Tirouvanziam R., Conrad C.K., Bottiglieri T., Herzenberg L.A., Moss R.B., Herzenberg L.A. (2006). High-dose oral N-acetylcysteine, a glutathione prodrug, modulates inflammation in cystic fibrosis. Proc. Natl. Acad. Sci. USA.

[103] Dauletbaev N., Fischer P., Aulbach B., Gross J., Kusche W., Thyroff-Friesinger U., Wagner T.O., Bargon J. (2009). A phase II study on safety and efficacy of high-dose N-acetylcysteine in patients with cystic fibrosis. Eur. J. Med. Res..

[104] Skov M., Pressler T., Lykkesfeldt J., Poulsen H.E., Jensen P.O., Johansen H.K., Qvist T., Kraemer D., Hoiby N., Ciofu O. (2015). The effect of short-term, high-dose oral N-acetylcysteine treatment on oxidative stress markers in cystic fibrosis patients with chronic P. aeruginosa infection—A pilot study. J. Cyst. Fibros..

[105] Conrad C., Lymp J., Thompson V., Dunn C., Davies Z., Chatfield B., Nichols D., Clancy J., Vender R., Egan M.E. (2015). Long-term treatment with oral N-acetylcysteine: Affects lung function but not sputum inflammation in cystic fibrosis subjects. A phase II randomized placebo-controlled trial. J. Cyst. Fibros..

[106] Griese M., Kappler M., Eismann C., Ballmann M., Junge S., Rietschel E., van Koningsbruggen-Rietschel S., Staab D., Rolinck-Werninghaus C., Mellies U. (2013). Inhalation treatment with glutathione in patients with cystic fibrosis. A randomized clinical trial. Am. J. Respir. Crit. Care Med..

[107] Suk J.S., Lai S.K., Boylan N.J., Dawson M.R., Boyle M.P., Hanes J. (2011). Rapid transport of muco-inert nanoparticles in cystic fibrosis sputum treated with N-acetyl cysteine. Nanomedicine.

[108] Hosoe H., Kaise T., Ohmori K., Isohama Y., Kai H., Takahama K., Miyata T. (1999). Mucolytic and antitussive effects of erdosteine. J. Pharm. Pharmacol..

[109] Hosoe H., Kaise T., Ohmori K. (2002). Effects on the reactive oxygen species of erdosteine and its metabolite in vitro. Arzneimittelforschung.

[110] Cazzola M., Page C., Rogliani P., Calzetta L., Matera M.G. (2020). Multifaceted Beneficial Effects of Erdosteine: More than a Mucolytic Agent. Drugs.

[111] Dechant K.L., Noble S. (1996). Erdosteine. Drugs.

[112] Marchioni C.F., Polu J.M., Taytard A., Hanard T., Noseda G., Mancini C. (1995). Evaluation of efficacy and safety of erdosteine in patients affected by chronic bronchitis during an infective exacerbation phase and receiving amoxycillin as basic treatment (ECOBES, European Chronic Obstructive Bronchitis Erdosteine Study). Int. J. Clin. Pharmacol. Ther..

[113] Aubier M., Berdah L. (1999). Multicenter, controlled, double-blind study of the efficacy and tolerance of Vectrine (erdostein) versus placebo in the treatment of stabilized chronic bronchitis with hypersecretion. Rev. Mal. Respir..

[114] Zheng J.P., Wen F.Q., Bai C.X., Wan H.Y., Kang J., Chen P., Yao W.Z., Ma L.J., Li X., Raiteri L. (2014). Twice daily N-acetylcysteine 600 mg for exacerbations of chronic obstructive pulmonary disease (PANTHEON): A randomised, double-blind placebo-controlled trial. Lancet Respir. Med..

[115] Zheng J.P., Kang J., Huang S.G., Chen P., Yao W.Z., Yang L., Bai C.X., Wang C.Z., Wang C., Chen B.Y. (2008). Effect of carbocisteine on acute exacerbation of chronic obstructive pulmonary disease (PEACE Study): A randomised placebo-controlled study. Lancet.

[116] Dal Negro R.W., Wedzicha J.A., Iversen M., Fontana G., Page C., Cicero A.F., Pozzi E., Calverley P.M.A. (2017). on behalf of the RESTORE group. Effect of erdosteine on the rate and duration of COPD exacerbations: The RESTORE study. Eur. Respir. J..

[117] Calverley P.M., Page C., Dal Negro R.W., Fontana G., Cazzola M., Cicero A.F., Pozzi E., Wedzicha J.A. (2019). Effect of Erdosteine on COPD Exacerbations in COPD Patients with Moderate Airflow Limitation. Int. J. Chronic Obstr. Pulm. Dis..

[118] Rogliani P., Matera M.G., Page C., Puxeddu E., Cazzola M., Calzetta L. (2019). Efficacy and safety profile of mucolytic/antioxidant agents in chronic obstructive pulmonary disease: A comparative analysis across erdosteine, carbocysteine, and N-acetylcysteine. Respir. Res..

[119] Howard W.W. (2015). Treating Bronchiectasis with Doxofylline and Erdosteine. U.S. Patent.

[120] Crisafulli E., Coletti O., Costi S., Zanasi E., Lorenzi C., Lucic S., Fabbri L.M., Bertini M., Clini E.M. (2007). Effectiveness of erdosteine in elderly patients with bronchiectasis and hypersecretion: A 15-day, prospective, parallel, open-label, pilot study. Clin. Ther..

[121] Chang A.B., Yerkovich S.T., Baines K.J., Burr L., Champion A., Chatfield M.D., Eg K.P., Goyal V., Marsh R.L., McCallum G.B. (2024). Erdosteine in children and adults with bronchiectasis (BETTER trial): Study protocol for a multicentre, double-blind, randomised controlled trial. BMJ Open Respir. Res..

[122] Yildirim Z., Sogut S., Odaci E., Iraz M., Ozyurt H., Kotuk M., Akyol O. (2003). Oral erdosteine administration attenuates cisplatin-induced renal tubular damage in rats. Pharmacol. Res..

[123] Miyake K., Kaise T., Hosoe H., Akuta K., Manabe H., Ohmori K. (1999). The effect of erdosteine and its active metabolite on reactive oxygen species production by inflammatory cells. Inflamm. Res..

[124] Braga P.C., Dal Sasso M., Zuccotti T. (2000). Assessment of the antioxidant activity of the SH metabolite I of erdosteine on human neutrophil oxidative bursts. Arzneimittelforschung.

[125] Moretti M., Marchioni C.F. (2007). An overview of erdosteine antioxidant activity in experimental research. Pharmacol. Res..

[126] Jang Y.Y., Song J.H., Shin Y.K., Han E.S., Lee C.S. (2003). Depressant effects of ambroxol and erdosteine on cytokine synthesis, granule enzyme release, and free radical production in rat alveolar macrophages activated by lipopolysaccharide. Pharmacol. Toxicol..

[127] Park J.S., Park M.Y., Cho Y.J., Lee J.H., Yoo C.G., Lee C.T., Lee S.M. (2016). Anti-inflammatory Effect of Erdosteine in Lipopolysaccharide-Stimulated RAW 264.7 Cells. Inflammation.

[128] Hayashi K., Hosoe H., Kaise T., Ohmori K. (2000). Protective effect of erdosteine against hypochlorous acid-induced acute lung injury and lipopolysaccharide-induced neutrophilic lung inflammation in mice. J. Pharm. Pharmacol..

[129] Demiralay R., Gursan N., Erdem H. (2006). Regulation of sepsis-induced apoptosis of pulmonary cells by posttreatment of erdosteine and N-aceylcysteine. Toxicology.

[130] Braga P.C., Dal Sasso M., Culici M., Verducci P., Lo Verso R., Marabini L. (2006). Effect of metabolite I of erdosteine on the release of human neutrophil elastase. Pharmacology.

[131] Basyigit I., Yildiz F., Cekmen M., Duman C., Bulut O. (2005). Effects of erdosteine on smoking-induced lipid peroxidation in healthy smokers. Drugs R D.

[132] Dal Negro R.W., Visconti M., Micheletto C., Tognella S. (2008). Changes in blood ROS, e-NO, and some pro-inflammatory mediators in bronchial secretions following erdosteine or placebo: A controlled study in current smokers with mild COPD. Pulm. Pharmacol. Ther..

[133] Dal Negro R., Visconti M., Trevisan F., Bertacco S., Micheletto C., Tognella S. (2008). Erdosteine enhances airway response to salbutamol in patients with mild-to-moderate COPD. Ther. Adv. Respir. Dis..

[134] Dal Negro R.W., Visconti M., Turco P. (2015). Efficacy of erdosteine 900 versus 600 mg/day in reducing oxidative stress in patients with COPD exacerbations: Results of a double blind, placebo-controlled trial. Pulm. Pharmacol. Ther..

[135] Dal Negro R.W., Visconti M. (2016). Erdosteine reduces the exercise-induced oxidative stress in patients with severe COPD: Results of a placebo-controlled trial. Pulm. Pharmacol. Ther..

[136] Moretti M., Fagnani S. (2015). Erdosteine reduces inflammation and time to first exacerbation postdischarge in hospitalized patients with AECOPD. Int. J. Chronic Obstr. Pulm. Dis..

[137] Cazzola M., Calzetta L., Page C., Rogliani P., Matera M.G. (2019). Thiol-Based Drugs in Pulmonary Medicine: Much More than Mucolytics. Trends Pharmacol. Sci..

[138] Pace E., Cerveri I., Lacedonia D., Paone G., Sanduzzi Zamparelli A., Sorbo R., Allegretti M., Lanata L., Scaglione F. (2022). Clinical Efficacy of Carbocysteine in COPD: Beyond the Mucolytic Action. Pharmaceutics.

[139] Song Y., Wang W., Xie Y., Xiang B., Huang X., Guan W., Zheng J. (2019). Carbocisteine inhibits the expression of Muc5b in COPD mouse model. Drug Des. Dev. Ther..

[140] Havez R., Degand P., Roussel P., Randoux A. (1970). Biochemical mode of action of cysteine by-products on the bronchial mucus. Poumon Coeur.

[141] Ishibashi Y., Takayama G., Inouye Y., Taniguchi A. (2010). Carbocisteine normalizes the viscous property of mucus through regulation of fucosylated and sialylated sugar chain on airway mucins. Eur. J. Pharmacol..

[142] Lopez-Vidriero M.T., Reid L. (1978). Chemical markers of mucous and serum glycoproteins and their relation to viscosity in mucoid and purulent sputum from various hypersecretory diseases. Am. Rev. Respir. Dis..

[143] Colombo B., Turconi P., Daffonchio L., Fedele G., Omini C., Cremaschi D. (1994). Stimulation of Cl- secretion by the mucoactive drug S-carboxymethylcysteine-lysine salt in the isolated rabbit trachea. Eur. Respir. J..

[144] Ikeuchi Y., Kogiso H., Hosogi S., Tanaka S., Shimamoto C., Matsumura H., Inui T., Marunaka Y., Nakahari T. (2019). Carbocisteine stimulated an increase in ciliary bend angle via a decrease in [Cl^−^]_i_ in mouse airway cilia. Pflug. Arch..

[145] Inui T.A., Murakami K., Yasuda M., Hirano S., Ikeuchi Y., Kogiso H., Hosogi S., Inui T., Marunaka Y., Nakahari T. (2019). Ciliary beating amplitude controlled by intracellular Cl^−^ and a high rate of CO_2_ production in ciliated human nasal epithelial cells. Pflug. Arch..

[146] Garavaglia M.L., Bononi E., Dossena S., Mondini A., Bazzini C., Lanata L., Balsamo R., Bagnasco M., Conese M., Botta G. (2008). S-CMC-Lys protective effects on human respiratory cells during oxidative stress. Cell. Physiol. Biochem..

[147] Guizzardi F., Rodighiero S., Binelli A., Saino S., Bononi E., Dossena S., Garavaglia M.L., Bazzini C., Botta G., Conese M. (2006). S-CMC-Lys-dependent stimulation of electrogenic glutathione secretion by human respiratory epithelium. J. Mol. Med..

[148] Brandolini L., Allegretti M., Berdini V., Cervellera M.N., Mascagni P., Rinaldi M., Melillo G., Ghezzi P., Mengozzi M., Bertini R. (2003). Carbocysteine lysine salt monohydrate (SCMC-LYS) is a selective scavenger of reactive oxygen intermediates (ROIs). Eur. Cytokine Netw..

[149] Yoshida M., Nakayama K., Yasuda H., Kubo H., Kuwano K., Arai H., Yamaya M. (2009). Carbocisteine inhibits oxidant-induced apoptosis in cultured human airway epithelial cells. Respirology.

[150] Wang W., Guan W.J., Huang R.Q., Xie Y.Q., Zheng J.P., Zhu S.X., Chen M., Zhong N.S. (2016). Carbocisteine attenuates TNF-alpha-induced inflammation in human alveolar epithelial cells in vitro through suppressing NF-kappaB and ERK1/2 MAPK signaling pathways. Acta Pharmacol. Sin..

[151] Alibasic E., Skopljak A., Cengic A., Krstovic G., Trifunovic N., Catic T., Kapo B., Mehic M., Hadzimuratovic A. (2017). Efficacy of carbocisteine in the treatment of chronic obstructive pulmonary disease and impact on the quality of life. Med. Glas..

[152] Grillage M., Barnard-Jones K. (1985). Long-term oral carbocisteine therapy in patients with chronic bronchitis. A double blind trial with placebo control. Br. J. Clin. Pract..

[153] Allegra L., Cordaro C.I., Grassi C. (1996). Prevention of acute exacerbations of chronic obstructive bronchitis with carbocysteine lysine salt monohydrate: A multicenter, double-blind, placebo-controlled trial. Respiration.

[154] Esposito A., Valentino M.R., Bruzzese D., Bocchino M., Ponticiello A., Stanziola A., Sanduzzi A. (2016). Effect of CArbocisteine in Prevention of exaceRbation of chronic obstructive pulmonary disease (CAPRI study): An observational study. Pulm. Pharmacol. Ther..

[155] Zeng Z., Yang D., Huang X., Xiao Z. (2017). Effect of carbocisteine on patients with COPD: A systematic review and meta-analysis. Int. J. Chronic Obstr. Pulm. Dis..

[156] Minov J., Stoleski S., Petrova T., Vasilevska K., Mijakoski D., Karadzinska-Bislimovska J. (2019). Effects of a Long-Term Use of Carbocysteine on Frequency and Duration of Exacerbations in Patients with Bronchiectasis. Open Access Maced. J. Med. Sci..

[157] Caramia G., Gagliardini R., Ruffini E., Osimani P., Nobilini A. (1995). The management of cystic fibrosis with carbocysteine lysine salt: Single-blind comparative study with ambroxol hydrochloride. J. Int. Med. Res..

[158] Tam J., Nash E.F., Ratjen F., Tullis E., Stephenson A. (2013). Nebulized and oral thiol derivatives for pulmonary disease in cystic fibrosis. Cochrane Database Syst. Rev..

[159] Hooper C., Calvert J. (2008). The role for S-carboxymethylcysteine (carbocisteine) in the management of chronic obstructive pulmonary disease. Int. J. Chronic Obstr. Pulm. Dis..

[160] Carpagnano G.E., Resta O., Foschino-Barbaro M.P., Spanevello A., Stefano A., Di Gioia G., Serviddio G., Gramiccioni E. (2004). Exhaled Interleukine-6 and 8-isoprostane in chronic obstructive pulmonary disease: Effect of carbocysteine lysine salt monohydrate (SCMC-Lys). Eur. J. Pharmacol..

[161] Ferraro M., Di Vincenzo S., Sangiorgi C., Leto Barone S., Gangemi S., Lanata L., Pace E. (2022). Carbocysteine Modifies Circulating miR-21, IL-8, sRAGE, and fAGEs Levels in Mild Acute Exacerbated COPD Patients: A Pilot Study. Pharmaceuticals.

[162] Zanasi A., Mazzolini M., Kantar A. (2017). A reappraisal of the mucoactive activity and clinical efficacy of bromhexine. Multidiscip. Respir. Med..

[163] Bhagat A., Rachana (2018). Bromhexine: A Comprehensive Review. Int. J. Biol. Med. Res..

[164] Sonawane K.D., Barale S.S., Dhanavade M.J., Waghmare S.R., Nadaf N.H., Kamble S.A., Mohammed A.A., Makandar A.M., Fandilolu P.M., Dound A.S. (2021). Structural insights and inhibition mechanism of TMPRSS2 by experimentally known inhibitors Camostat mesylate, Nafamostat and Bromhexine hydrochloride to control SARS-coronavirus-2: A molecular modeling approach. Inform. Med. Unlocked.

[165] Gil J., Thurnheer U. (1971). Morphometric evaluation of ultrastructural changes in type II alveolar cells of rat lung produced by bromhexine. Respiration.

[166] Valenti S., Marenco G. (1989). Italian multicenter study on the treatment of chronic obstructive lung disease with bromhexine. A double-blind placebo-controlled trial. Respiration.

[167] Flavell Matts S., Zorbala-Mallios H., Southgate J.J.C.T.J. (1973). Sputum fibre systems in exacerbations of longstanding pulmonary disease. A comparison of antibiotics and bromhexine (Bisolvan). Clin. Trials J..

[168] Nesswetha W. (1967). Criteria of drug testing in industrial practice, demonstrated by a cough remedy. Arzneimittelforschung.

[169] Burgi H. (1974). Changes in the fibre system and viscosity of the sputum of bronchitis during treatment with bromhexine and guaiphenesin (guaiacol glyceryl ether). Scand. J. Respir. Dis..

[170] Thomson M.L., Pavia D., Gregg I., Stark J.E. (1974). Bromhexine and mucociliary clearance in chronic bronchitis. Br. J. Dis. Chest.

[171] Gent M., Knowlson P.A., Rime F.J. (1969). Effect of bromhexine on ventilatory capacity in patients with a variety of chest diseases. Lancet.

[172] Hamilton W.F., Palmer K.N., Gent M. (1970). Expectorant action of bromhexine in chronic obstructive bronchitis. Br. Med. J..

[173] Christensen F., Kjer J., Ryskjaer S., Arseth-Hansen P. (1970). Bromhexine in chronic bronchitis. Br. Med. J..

[174] Papadopoulou E., Hansel J., Lazar Z., Kostikas K., Tryfon S., Vestbo J., Mathioudakis A.G. (2023). Mucolytics for acute exacerbations of chronic obstructive pulmonary disease: A meta-analysis. Eur. Respir. Rev..

[175] Langlands J.H. (1970). Double-blind clinical trial of bromhexine as a mucolytic drug in chronic bronchitis. Lancet.

[176] Maesen F.P., Davies B.I., Brouwers J., Rubingh G. (1982). Erythromycin and bromhexine in acute exacerbations of chronic bronchitis. A study on sputum penetration and clinical effectiveness. Eur. J. Respir. Dis..

[177] Olivieri D., Ciaccia A., Marangio E., Marsico S., Todisco T., Del Vita M. (1991). Role of bromhexine in exacerbations of bronchiectasis. Double-blind randomized multicenter study versus placebo. Respiration.

[178] Felix K., Pairet M., Zimmermann R. (1996). The antioxidative activity of the mucoregulatory agents: Ambroxol, bromhexine and N-acetyl-L-cysteine. A pulse radiolysis study. Life Sci..

[179] Malerba M., Ragnoli B. (2008). Ambroxol in the 21st century: Pharmacological and clinical update. Expert Opin. Drug Metab. Toxicol..

[180] Ahmadi E., Afrooghe A., Soltani Z.E., Elahi M., Shayan M., Ohadi M.A.D., Dehpour A.R. (2024). Beyond the lungs: Exploring diverse applications of bromhexine and ambroxol. Life Sci..

[181] Olivieri D., Zavattini G., Tomasini G., Daniotti S., Bonsignore G., Ferrara G., Carnimeo N., Chianese R., Catena E., Marcatili S. (1987). Ambroxol for the prevention of chronic bronchitis exacerbations: Long-term multicenter trial. Protective effect of ambroxol against winter semester exacerbations: A double-blind study versus placebo. Respiration.

[182] Matthys H., de Mey C., Carls C., Rys A., Geib A., Wittig T. (2000). Efficacy and tolerability of myrtol standardized in acute bronchitis. A multi-centre, randomised, double-blind, placebo-controlled parallel group clinical trial vs. cefuroxime and ambroxol. Arzneimittelforschung.

[183] Malerba M., Ponticiello A., Radaeli A., Bensi G., Grassi V. (2004). Effect of twelve-months therapy with oral ambroxol in preventing exacerbations in patients with COPD. Double-blind, randomized, multicenter, placebo-controlled study (the AMETHIST Trial). Pulm. Pharmacol. Ther..

[184] Kantar A., Klimek L., Cazan D., Sperl A., Sent U., Mesquita M. (2020). An overview of efficacy and safety of ambroxol for the treatment of acute and chronic respiratory diseases with a special regard to children. Multidiscip. Respir. Med..

[185] Gupta P.R. (2010). Ambroxol—Resurgence of an old molecule as an anti-inflammatory agent in chronic obstructive airway diseases. Lung India.

[186] Cerutti P., Kapanci Y. (1979). Effects of metabolite VIII of bromexine (Na 872) on type II epithelium of the lung: An experimental and morphological study with reference to surfactant secretion. Respiration.

[187] Elemer G., Kapanci Y. (1983). Effect of Ambroxol on pneumocyte type II cell. A morphological and biochemical study. Curr. Probl. Clin. Biochem..

[188] Stockley R.A., Shaw J., Burnett D. (1988). Effect of ambroxol of neutrophil chemotaxis in vitro. Agents Actions.

[189] Zhang S.J., Jiang J.X., Ren Q.Q., Jia Y.L., Shen J., Shen H.J., Lin X.X., Lu H., Xie Q.M. (2016). Ambroxol inhalation ameliorates LPS-induced airway inflammation and mucus secretion through the extracellular signal-regulated kinase 1/2 signaling pathway. Eur. J. Pharmacol..

[190] Abdelaziz A.A., Abo-Kamar A.M., Ashour A.E., Shaldam M.A., Elekhnawy E. (2024). Unveiling the antibacterial action of ambroxol against Staphylococcus aureus bacteria: In vitro, in vivo, and in silico investigation. BMC Microbiol..

[191] Gillissen A., Bartling A., Schoen S., Schultze-Werninghaus G. (1997). Antioxidant function of ambroxol in mononuclear and polymorphonuclear cells in vitro. Lung.

[192] Ottonello L., Arduino N., Bertolotto M., Dapino P., Mancini M., Dallegri F. (2003). In vitro inhibition of human neutrophil histotoxicity by ambroxol: Evidence for a multistep mechanism. Br. J. Pharmacol..

[193] Lee C.S., Jang Y.Y., Song J.S., Song J.H., Han E.S. (2002). Ambroxol inhibits peroxynitrite-induced damage of alpha1-antiproteinase and free radical production in activated phagocytic cells. Pharmacol. Toxicol..

[194] Ricciardolo F.L., Sorbello V., Benedetto S., Paleari D. (2015). Effect of Ambroxol and Beclomethasone on Lipopolysaccharide-Induced Nitrosative Stress in Bronchial Epithelial Cells. Respiration.

[195] Ollier C., Sent U., Mesquita M., Michel M.C. (2020). Pharmacokinetics of Ambroxol Sustained Release (Mucosolvan((R)) Retard) Compared with Other Formulations in Healthy Volunteers. Pulm. Ther..

[196] Zheng Z., Yang K., Liu N., Fu X., He H., Chen H., Xu P., Wang J., Liu M., Tang Y. (2023). Evaluation of safety and efficacy of inhaled ambroxol in hospitalized adult patients with mucopurulent sputum and expectoration difficulty. Front. Med..

[197] Li Z. (2021). The effect of adjuvant therapy with ambroxol hydrochloride in elderly chronic obstructive pulmonary disease patients. Am. J. Transl. Res..

[198] Anil A.A., Rajesh D., Krishnan A.L., Arun C., Thomas R., Chandra P., Haridas N. (2024). Evaluating the mucolytic effectiveness of Ambroxol and N-Acetylcysteine in patients with Acute exacerbation of chronic obstructive pulmonary disease. Clin. Epidemiol. Glob. Health.

[199] Ratjen F., Wonne R., Posselt H.G., Stover B., Hofmann D., Bender S.W. (1985). A double-blind placebo controlled trial with oral ambroxol and N-acetylcysteine for mucolytic treatment in cystic fibrosis. Eur. J. Pediatr..

[200] Cazan D., Klimek L., Sperl A., Plomer M., Kolsch S. (2018). Safety of ambroxol in the treatment of airway diseases in adult patients. Expert Opin. Drug Saf..

[201] Saito D., Suzuki C., Tanaka S., Hosogi S., Kawaguchi K., Asano S., Okamoto S., Yasuda M., Hirano S., Inui T. (2023). Ambroxol-enhanced ciliary beating via voltage-gated Ca^2+^ channels in mouse airway ciliated cells. Eur. J. Pharmacol..

[202] Gillissen A., Nowak D. (1998). Characterization of N-acetylcysteine and ambroxol in anti-oxidant therapy. Respir. Med..

[203] Roesch E.A., Rahmaoui A., Lazarus R.A., Konstan M.W. (2024). The continuing need for dornase alfa for extracellular airway DNA hydrolysis in the era of CFTR modulators. Expert Rev. Respir. Med..

[204] Hitchcock S.E., Carisson L., Lindberg U. (1976). Depolymerization of F-actin by deoxyribonuclease I. Cell.

[205] Vasconcellos C.A., Allen P.G., Wohl M.E., Drazen J.M., Janmey P.A., Stossel T.P. (1994). Reduction in viscosity of cystic fibrosis sputum in vitro by gelsolin. Science.

[206] Terlizzi V., Castellani C., Taccetti G., Ferrari B. (2022). Dornase alfa in Cystic Fibrosis: Indications, comparative studies and effects on lung clearance index. Ital. J. Pediatr..

[207] Sun F., Tai S., Lim T., Baumann U., King M. (2002). Additive effect of dornase alfa and Nacystelyn on transportability and viscoelasticity of cystic fibrosis sputum. Can. Respir. J..

[208] Fuchs H.J., Borowitz D.S., Christiansen D.H., Morris E.M., Nash M.L., Ramsey B.W., Rosenstein B.J., Smith A.L., Wohl M.E. (1994). Effect of aerosolized recombinant human DNase on exacerbations of respiratory symptoms and on pulmonary function in patients with cystic fibrosis. The Pulmozyme Study Group. N. Engl. J. Med..

[209] McCoy K., Hamilton S., Johnson C., for the Pulmozyme Study Group (1996). Effects of 12-week administration of dornase alfa in patients with advanced cystic fibrosis lung disease. Chest.

[210] Quan J.M., Tiddens H.A., Sy J.P., McKenzie S.G., Montgomery M.D., Robinson P.J., Wohl M.E., Konstan M.W. (2001). A two-year randomized, placebo-controlled trial of dornase alfa in young patients with cystic fibrosis with mild lung function abnormalities. J. Pediatr..

[211] Frederiksen B., Pressler T., Hansen A., Koch C., Høiby N. (2006). Effect of aerosolized rhDNase (Pulmozyme) on pulmonary colonization in patients with cystic fibrosis. Acta Paediatr..

[212] Jones A.P., Wallis C.E. (2003). Recombinant human deoxyribonuclease for cystic fibrosis. Cochrane Database Syst. Rev..

[213] Amin R., Subbarao P., Lou W., Jabar A., Balkovec S., Jensen R., Kerrigan S., Gustafsson P., Ratjen F. (2011). The effect of dornase alfa on ventilation inhomogeneity in patients with cystic fibrosis. Eur. Respir. J..

[214] Voldby C., Green K., Philipsen L., Sandvik R.M., Skov M., Buchvald F., Pressler T., Nielsen K.G. (2021). Withdrawal of dornase alfa increases ventilation inhomogeneity in children with cystic fibrosis. J. Cyst. Fibros..

[215] Rowe S.M., Hoover W., Solomon G.M., Sorscher E.J., Broaddus V.C., Mason R.J., Ernst J.D., King T.E., Lazarus S.C., Murray J.F., Nadel J.A., Slutsky A.S., Gotway M.B. (2016). 47—Cystic Fibrosis. Murray and Nadel’s Textbook of Respiratory Medicine.

[216] Henry R.L., Gibson P.G., Carty K., Cai Y., Francis J.L. (1998). Airway inflammation after treatment with aerosolized deoxyribonuclease in cystic fibrosis. Pediatr. Pulmonol..

[217] Ratjen F., Paul K., van Koningsbruggen S., Breitenstein S., Rietschel E., Nikolaizik W. (2005). DNA concentrations in BAL fluid of cystic fibrosis patients with early lung disease: Influence of treatment with dornase alpha. Pediatr. Pulmonol..

[218] Paul K., Rietschel E., Ballmann M., Griese M., Worlitzsch D., Shute J., Chen C., Schink T., Döring G., van Koningsbruggen S. (2004). Effect of treatment with dornase alpha on airway inflammation in patients with cystic fibrosis. Am. J. Respir. Crit. Care Med..

[219] Ratjen F., Hartog C.M., Paul K., Wermelt J., Braun J. (2002). Matrix metalloproteases in BAL fluid of patients with cystic fibrosis and their modulation by treatment with dornase alpha. Thorax.

[220] Konstan M.W., Ratjen F. (2012). Effect of dornase alfa on inflammation and lung function: Potential role in the early treatment of cystic fibrosis. J. Cyst. Fibros..

[221] Addante A., Raymond W., Gitlin I., Charbit A., Orain X., Scheffler A.W., Kuppe A., Duerr J., Daniltchenko M., Drescher M. (2023). A novel thiol-saccharide mucolytic for the treatment of muco-obstructive lung diseases. Eur. Respir. J..

[222] Yan X., Sha X. (2023). Nanoparticle-Mediated Strategies for Enhanced Drug Penetration and Retention in the Airway Mucosa. Pharmaceutics.

[223] Peng S., Wang W., Zhang R., Wu C., Pan X., Huang Z. (2024). Nano-Formulations for Pulmonary Delivery: Past, Present, and Future Perspectives. Pharmaceutics.

[224] Chen D., Liu J., Wu J., Suk J.S. (2021). Enhancing nanoparticle penetration through airway mucus to improve drug delivery efficacy in the lung. Expert Opin. Drug Deliv..

[225] Hami Z. (2021). A Brief Review on Advantages of Nano-based Drug Delivery Systems. Ann. Mil. Health Sci. Res..

[226] Rouillard K.R., Markovetz M.R., Kissner W.J., Boone W.L., Plott L.M., Hill D.B. (2023). Altering the viscoelastic properties of mucus-grown Pseudomonas aeruginosa biofilms affects antibiotic susceptibility. Biofilm.

[227] Staltari G., Biasin A., Grassi L., Gerin F., Maschio M., Confalonieri M., Grassi G., Grassi M., Abrami M. (2023). Rheological and Low Field NMR Characterisation of Cystic Fibrosis Patient’s Sputum. Chem. Biochem. Eng. Q..

[228] Flory P.J. (1953). Principles of Polymer Chemistry.

[229] Mezzasalma S.A., Abrami M., Grassi G., Grassi M. (2025). Towards a universal size distribution in a polymer network. Implications for drug delivery and plasmonic nanoparticle transport phenomena in polysaccharide and synthetic hydrogels. Int. J. Biol. Macromol..

[230] Schurz J. (1991). Rheology of polymer solutions of the network type. Prog. Polym. Sci..

[231] Duncan G.A., Jung J., Joseph A., Thaxton A.L., West N.E., Boyle M.P., Hanes J., Suk J.S. (2016). Microstructural alterations of sputum in cystic fibrosis lung disease. J. Clin. Investig. Insight.

[232] Amblard F., Maggs A.C., Yurke B., Pargellis A., Leibler S. (1996). Subdiffusion and Anomalous Local Viscoelasticity in Actin Networks. Phys. Rev. Lett..

[233] Mason T.G., Weitz D.A. (1995). Optical measurements of frequency-dependent linear viscoelastic moduli of complex fluids. Phys. Rev. Lett..

[234] Ziemann F., Radler J., Sackmann E. (1994). Local measurements of viscoelastic moduli of entangled actin networks using an oscillating magnetic bead micro-rheometer. Biophys. J..

[235] Mason T., Dhople A., Wirtz D. (1996). Concentrated DNA Rheology and Microrheology. MRS Proc..

[236] Gennes P.G.D. (1979). Scaling Concepts in Polymer Physics.

[237] Dawson M., Wirtz D., Hanes J. (2003). Enhanced viscoelasticity of human cystic fibrotic sputum correlates with increasing microheterogeneity in particle transport. J. Biol. Chem..

[238] Kirch J., Schneider A., Abou B., Hopf A., Schaefer U.F., Schneider M., Schall C., Wagner C., Lehr C.M. (2012). Optical tweezers reveal relationship between microstructure and nanoparticle penetration of pulmonary mucus. Proc. Natl. Acad. Sci. USA.

[239] Abrami M., Maschio M., Conese M., Confalonieri M., Gerin F., Dapas B., Farra R., Adrover A., Torelli L., Ruaro B. (2021). Combined use of rheology and portable low-field NMR in cystic fibrosis patients. Respir. Med..

[240] Abrami M., Maschio M., Conese M., Confalonieri M., Salton F., Gerin F., Dapas B., Farra R., Adrover A., Milcovich G. (2022). Effect of chest physiotherapy on cystic fibrosis sputum nanostructure: An experimental and theoretical approach. Drug Deliv. Transl. Res..

[241] Brownstein K.R., Tarr C.E. (1979). Importance of classical diffusion in NMR studies of water in biological cells. Phys. Rev. A.

[242] Chui M.M., Phillips R.J., McCarthy M.J. (1995). Measurement of the Porous Microstructure of Hydrogels by Nuclear Magnetic Resonance. J. Colloid Interface Sci..

[243] Abrami M., Ascenzioni F., Di Domenico E.G., Maschio M., Ventura A., Confalonieri M., Di Gioia S., Conese M., Dapas B., Grassi G. (2018). A novel approach based on low-field NMR for the detection of the pathological components of sputum in cystic fibrosis patients. Magn. Reson. Med..

[244] Abrami M., Maschio M., Conese M., Confalonieri M., Di Gioia S., Gerin F., Dapas B., Tonon F., Farra R., Murano E. (2020). Use of low field nuclear magnetic resonance to monitor lung inflammation and the amount of pathological components in the sputum of cystic fibrosis patients. Magn. Reson. Med..

[245] Scherer G.W. (1994). Hydraulic radius and mesh size of gels. J. Sol-Gel Sci. Technol..

